# Physiologically-Based Pharmacokinetic (PBPK) Modeling of Buprenorphine in Adults, Children and Preterm Neonates

**DOI:** 10.3390/pharmaceutics12060578

**Published:** 2020-06-23

**Authors:** Lukas Kovar, Christina Schräpel, Dominik Selzer, Yvonne Kohl, Robert Bals, Matthias Schwab, Thorsten Lehr

**Affiliations:** 1 Department of Clinical Pharmacy, Saarland University, 66123 Saarbrücken, Germany; lukas.kovar@uni-saarland.de (L.K.); christina.schraepel@uni-saarland.de (C.S.); dominik.selzer@uni-saarland.de (D.S.); 2 Dr. Margarete Fischer-Bosch-Institute of Clinical Pharmacology, 70376 Stuttgart, Germany; Matthias.Schwab@ikp-stuttgart.de; 3 Fraunhofer Institute for Biomedical Engineering IBMT, 66280 Sulzbach, Germany; yvonne.kohl@ibmt.fraunhofer.de; 4 Department of Internal Medicine V, Saarland University, 66421 Homburg, Germany; robert.bals@uks.eu; 5 Departments of Clinical Pharmacology, and Pharmacy and Biochemistry, University Tübingen, 72076 Tübingen, Germany

**Keywords:** physiologically based pharmacokinetic (PBPK) modeling, buprenorphine, drug-drug interaction (DDI), norbuprenorphine, pediatric scaling, pharmacokinetics

## Abstract

Buprenorphine plays a crucial role in the therapeutic management of pain in adults, adolescents and pediatric subpopulations. However, only few pharmacokinetic studies of buprenorphine in children, particularly neonates, are available as conducting clinical trials in this population is especially challenging. Physiologically-based pharmacokinetic (PBPK) modeling allows the prediction of drug exposure in pediatrics based on age-related physiological differences. The aim of this study was to predict the pharmacokinetics of buprenorphine in pediatrics with PBPK modeling. Moreover, the drug-drug interaction (DDI) potential of buprenorphine with CYP3A4 and P-glycoprotein perpetrator drugs should be elucidated. A PBPK model of buprenorphine and norbuprenorphine in adults has been developed and scaled to children and preterm neonates, accounting for age-related changes. One-hundred-percent of the predicted AUC_last_ values in adults (geometric mean fold error (GMFE): 1.22), 90% of individual AUC_last_ predictions in children (GMFE: 1.54) and 75% in preterm neonates (GMFE: 1.57) met the 2-fold acceptance criterion. Moreover, the adult model was used to simulate DDI scenarios with clarithromycin, itraconazole and rifampicin. We demonstrate the applicability of scaling adult PBPK models to pediatrics for the prediction of individual plasma profiles. The novel PBPK models could be helpful to further investigate buprenorphine pharmacokinetics in various populations, particularly pediatric subgroups.

## 1. Introduction

Buprenorphine is a partial agonist of the µ-opioid receptor with an analgesic potency 25 to 100 times greater compared with that of morphine [1,2]. As such, buprenorphine plays a crucial role in the therapeutic management of pain in adults and adolescents, which is suggested among others in a recent guideline on cancer pain management of the World Health Organization (WHO) [2]. Furthermore, in recent years the use of buprenorphine has become widespread in pediatrics with indications ranging from postoperative analgesia to chronic pain in palliative care [3,4].

Buprenorphine displays a ceiling effect in adults, in which escalating doses do not cause additional respiratory depression [5,6]. However, this effect does not seem to apply to young children [7,8]. As a result, buprenorphine-related serious adverse reactions (ADR) up to fatal events have been reported, especially in young children, as well as single cases of accidental poisoning due to improperly stored buprenorphine drug products [7,9,10].

As a consequence, a recent meta-analysis by Vicencio-Rosas and colleagues pointed out the need of further research activities on buprenorphine in pediatric populations with particular focus on pharmacokinetic and pharmacodynamic issues [3]. Among others, their goals should be to allow researchers to develop dosage schemes and minimize the risk of ADR [3]. However, pediatric studies are difficult to conduct and are accompanied by numerous ethical challenges, many of which are unique to pediatrics, especially newborns [11]. Physiologically-based pharmacokinetic (PBPK) modeling in pediatrics has shown to be useful for the optimization of clinical study designs, the prediction of starting doses for children and the assessment of potential drug-drug interactions (DDIs) [12,13,14,15,16].

Compared to most other opioid receptor agonists, the potential for drug abuse and drug overdose in adults is lower due to buprenorphine’s partial agonism and its ceiling effect in the adult population [9,17]. Hence, buprenorphine has successfully been used in the treatment of opioid use disorders (OUD) and is helping combat the current opioid epidemic [18,19]. However, the increase in buprenorphine prescriptions has also been associated with illicit usage, raising concerns about the potential of misuse and diversion [9,20].

A major metabolic route of elimination of buprenorphine represents the metabolism to the active metabolite, norbuprenorphine, mainly through the cytochrome P450 (CYP) 3A4 enzyme, an enzyme with a high DDI potential [21,22]. As a result, buprenorphine and norbuprenorphine plasma levels can be affected by CYP3A4 inhibitors and inducers [21,23]. Recently conducted DDI studies with CYP3A4 perpetrator drugs have shown significant changes in buprenorphine plasma concentrations after specific oral and sublingual administration scenarios [24,25,26]. Still, the clinical relevance of other DDIs with frequently used perpetrator drugs (e.g., clarithromycin or itraconazole) and the impact of the inhibition and/or induction of the drug transporter P-glycoprotein (P-gp) remain unclear [23]. PBPK modeling has shown to be a powerful tool in predicting and simulating DDI scenarios and drug concentrations at specific target sites. Moreover, PBPK models are useful to elucidate transporter proteins and their contribution to drug disposition [22,27,28,29].

The objectives of this study were (1) to establish and evaluate a whole-body parent-metabolite intravenous PBPK model of buprenorphine and norbuprenorphine in adults, (2) to scale the adult PBPK model to pediatrics for the assessment of plasma concentration-time profiles, and (3) to use the developed adult PBPK model for the evaluation of DDIs with frequently used CYP3A4 and P-gp perpetrator drugs that have not been investigated yet. The novel PBPK models are publicly available in the Open Systems Pharmacology (OSP) repository as clinical research tools to support the design of clinical trials in specific populations as well as the development of novel drug formulations. The Supplementary Materials serve as a comprehensive reference manual including detailed documentation of the model performance assessment.

## 2. Materials and Methods

### 2.1. Software

The PBPK models were developed with the PK-Sim^®^ modeling software (version 8.0, part of the OSP Suite). Model input parameter optimization was accomplished using the Monte Carlo algorithm implemented in PK-Sim^®^. Clinical data in scientific literature were digitized using GetData Graph Digitizer version 2.26.0.20 (S. Fedorov) according to best practices [30]. Allometric scaling was performed in NONMEM^®^ (Version 7.4.3), pharmacokinetic (PK) parameter analyses and graphics with the R programming language version 3.6.1 (R Foundation for Statistical Computing, Vienna, Austria) and R Studio^®^ version 1.2.5019 (R Studio, Inc., Boston, MA, USA).

### 2.2. PBPK Parent-Metabolite Model Building in Adults

In agreement with pediatric PBPK model development workflows, first, an adult PBPK model was built and subsequently evaluated with observed plasma profiles to promote confidence in the parametrization of the PBPK model, before the model was scaled to pediatric populations [12,31,32,33]. For the building of the adult parent-metabolite PBPK model of buprenorphine and norbuprenorphine, an extensive literature search was performed to obtain information on (a) physicochemical properties, (b) distribution, metabolism and excretion processes of the two modeled compounds as well as (c) clinical studies of intravenous administration of buprenorphine. The gathered information was used to implement relevant transport proteins and enzymes involved in distribution, metabolism and excretion processes and to inform drug-dependent model input parameters. The plasma profiles of the identified clinical studies were digitized and split into an internal training and an external test dataset. The selection of studies for the internal dataset was guided by the information contained in the different studies (i.e., dosing regimens, frequent as well as early and late sampling, measurements of norbuprenorphine, measurements of arterial plasma concentrations, etc.). To obtain values for model input parameters, which could not be adequately obtained from literature, parameter estimation was performed by fitting the parent-metabolite model to the training dataset. The external test dataset was used for model evaluation.

**Figure 1:**
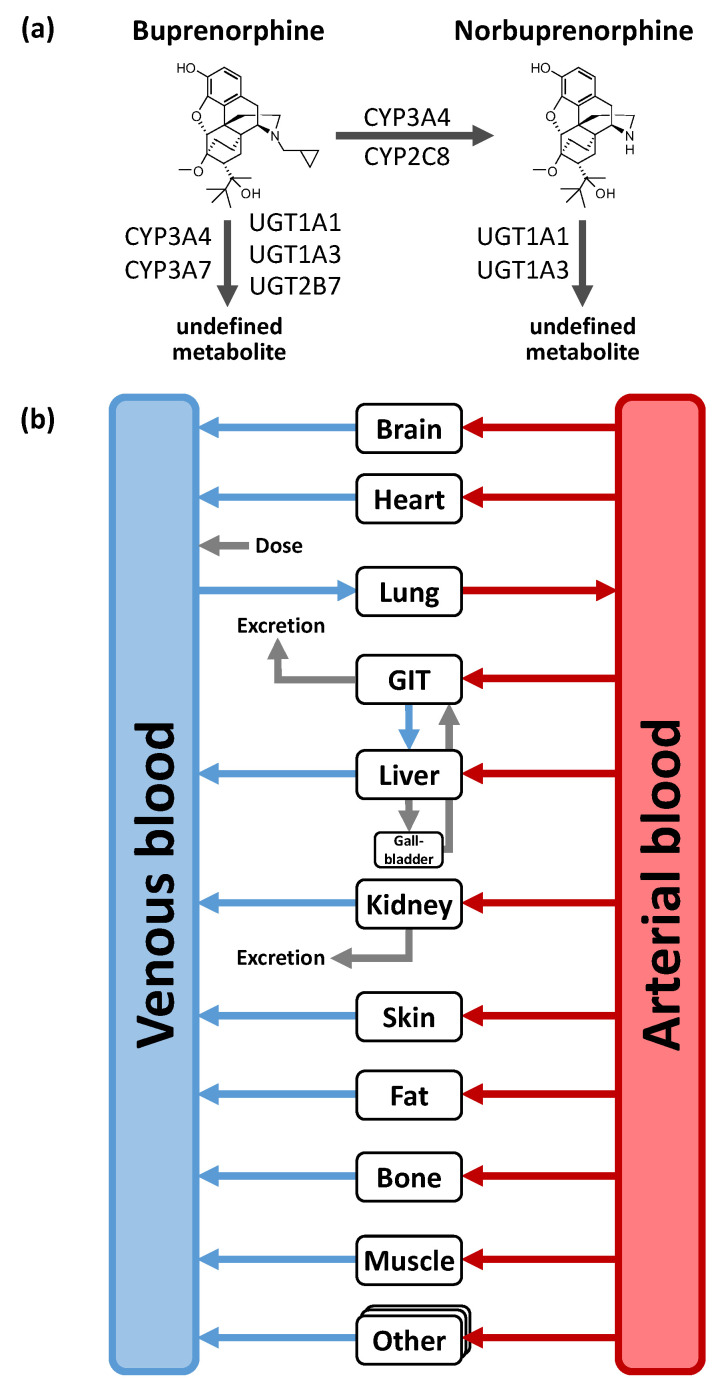
Implemented metabolic processes for buprenorphine and norbuprenorphine (**a**) and structural overview of the physiologically-based pharmacokinetic (PBPK) model (**b**). Boxes indicate compartments, black lines indicate metabolic processes, blue, grey and red lines denote in-/out-flows. CYP: cytochrome P450, GIT: gastrointestinal tract, UGT: uridine 5ʹ-diphospho-glucuronosyltransferase.

Distribution and elimination processes including CYP and uridine 5′-diphospho-glucuronosyltransferase (UGT) enzymes as well as drug transporter were implemented according to the literature [21,34,35]. For the buprenorphine model, these are (1) metabolism of buprenorphine to a major active metabolite norbuprenorphine through CYP3A4 and CYP2C8; (2) metabolism pathways metabolizing buprenorphine to other non-specified metabolites through CYP3A4, CYP3A7, UGT1A1, UGT1A3, and UGT2B7; as well as (3) renal excretion through glomerular filtration. For the norbuprenorphine model, metabolism through UGT1A1 and UGT1A3 as well as renal clearance by glomerular filtration and tubular secretion through the transport protein P-gp were implemented [36,37]. Figure 1 shows a structural overview of the PBPK model including the implemented metabolic processes of buprenorphine and norbuprenorphine. Tissue expression distribution of the implemented enzymes was informed by the PK-Sim^®^ expression database [38]. For detailed supplementary information on PBPK model building see Section 1 in the Supplementary Materials.

### 2.3. Pediatric Scaling and Model Applications

After the building and evaluation of the adult PBPK model, the model was scaled to the administration of buprenorphine in children and preterm neonates for a priori predictions of the PK in the two pediatric populations. For this, the adult virtual populations were replaced by pediatric populations. These virtual pediatric populations were based on the patient characteristics of two included pediatric clinical trials with children and preterm neonates, respectively. As a result, both anatomic and physiological parameters as well as enzyme tissue concentrations were scaled to values of the respective target population accounting for age-related changes such as size and composition of tissue compartments, protein binding and maturation of elimination processes. Information on the ontogeny functions for enzymes can be found in [39] and in Table S1 of the Supplementary Materials. For scaling the fraction unbound of buprenorphine to children and preterm neonates, the method of McNamara and Alcorn for alpha-1-acid glycoprotein was applied [40,41]. The extrapolated PBPK model was subsequently used to predict 22 individual plasma concentration-time profiles in children and preterm neonates. To compare the outcome of the PK predictions using the pediatric PBPK models, a classical allometric scaling approach, as described by Tod et al., was used [42]. For detailed information on the allometric scaling, see Section 3 in the Supplementary Materials.

Additionally, the adult PBPK model was used to assess the DDI potential of the CYP and UGT substrate buprenorphine with the three perpetrator drugs clarithromycin, itraconazole and rifampicin. While itraconazole and its metabolites inhibit both CYP3A4 and P-gp competitively, clarithromycin is a mechanism-based inhibitor of the CYP3A4 enzyme and also competitively inhibits P-gp, yet with a much higher inhibition constant (K_i_) [22]. In contrast, rifampicin both inhibits and induces the CYP2C8, CYP3A4, UGT1A1, and UGT1A3 enzymes as well as the P-gp efflux transporter [22,43,44,45,46,47,48,49,50]. For the simulation of buprenorphine plasma profiles in the DDI scenarios, the buprenorphine model was coupled with recently published PBPK models of clarithromycin, itraconazole and rifampicin [22]. The rifampicin model was further extended with information on the CYP2C8, UGT1A1 and UGT1A3 induction and inhibition processes. Detailed information is provided in Section 2 of the Supplementary Materials.

### 2.4. PBPK Model Evaluation

Adult and pediatric PBPK model performances were evaluated with several methods. Predicted and observed areas under the plasma concentration-time curve from the first to the last data point (AUC_last_) and maximum plasma concentration (C_max_) values as well as the predicted plasma concentrations and their respective values observed were compared in goodness-of-fit plots. Moreover, buprenorphine and norbuprenorphine plasma concentration-time profiles observed both from adult and pediatric studies were visually compared to the plasma profiles predicted with the PBPK models. To estimate the variability of plasma profiles, virtual populations of 100 individuals were generated representing the corresponding clinical trial population. For detailed information on virtual populations see Section 1.2 in the Supplementary Materials. Individual plasma concentration-time profiles including the corresponding individual demographics were available in one study [51]. Here, populations of 100 individuals with the same demographics were used for simulations only allowing variability in the expression of the implemented enzymes and transporters. Population predictions were plotted as geometric mean with geometric standard deviation. When individual concentration-time datasets were available but demographic values could not be matched to the specific profile, median with 90% population prediction intervals were plotted. The sensitivity of the final PBPK models to single parameter changes (local sensitivity analysis) was investigated with PK-Sim^®^. Furthermore, two quantitative performance measures were calculated: the mean relative deviation (MRD) of the predicted plasma concentrations for each single plasma profile as well as the geometric mean fold errors (GMFE) of AUC_last_ and C_max_ ratios, respectively (for detailed information including equations please refer to Section 4.3 in the Supplementary Materials). C_max_ values were calculated only for intravenous long-term infusions and norbuprenorphine metabolite. Conclusively, the percentage of model-predicted concentrations falling within 2-fold of the corresponding observed concentrations was examined in addition to the mentioned evaluation measures above.

The DDI effects were evaluated by comparing plasma concentration-time profiles of buprenorphine and norbuprenorphine after buprenorphine administration alone (control) and plasma profiles during concomitant use with the DDI perpetrator (inhibition/induction). Additionally, the corresponding predicted AUC ratios (AUC_inhibition/induction, predicted_/AUC_control, predicted_) were calculated. Since observed data of a DDI clinical trial with rifampicin was available, the AUC ratio predicted was also compared to the AUC ratio observed in the respective DDI study.

## 3. Results

### 3.1. PK Data for PBPK Model Development and Pediatric Scaling

After a comprehensive literature search, eight PK studies in adults with 17 different treatment blocks after intravenous administration of buprenorphine were identified. Two of these studies were performed in an elderly population, one was a DDI study with rifampicin as the perpetrator drug. The dataset encompasses wide mean age and dose ranges with 21 to 67.5 years and 0.3 to 16 mg buprenorphine, respectively. In six treatment blocks, norbuprenorphine plasma concentrations were reported. All plasma concentration-time profiles were digitized and split into an internal training (n = 7 profiles) and an external test dataset (n = 16 profiles). The internal training dataset was complemented with information on the fraction of buprenorphine metabolized to norbuprenorphine, fraction of buprenorphine excreted unchanged in urine, and fraction of dose excreted in urine as norbuprenorphine [35,52,53]. For the evaluation of the PBPK model predictions in pediatrics, two clinical trials investigating buprenorphine plasma concentrations in both children (age: 4.6–7.5 years) and preterm neonates (27–34 weeks postmenstrual age) were located and the data digitized. An overview of the included clinical studies, comprising study characteristics and dosing regimens, is shown in Table 1.

### 3.2. Adult PBPK Model Building and Evaluation

**Figure 2:**
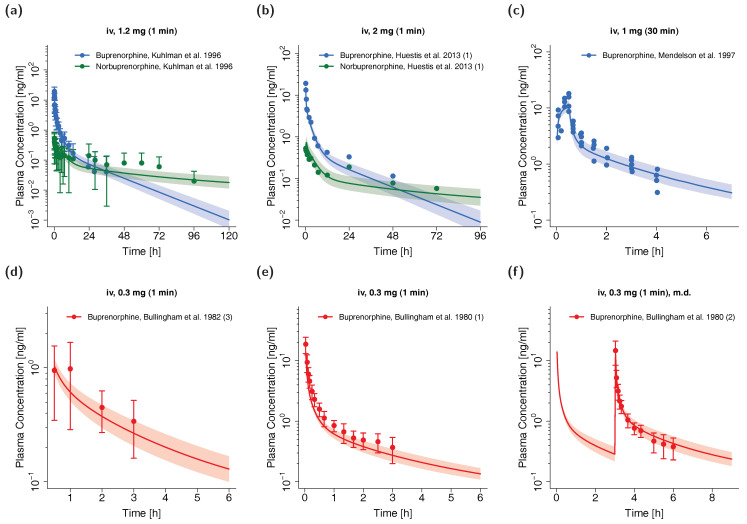
Buprenorphine (blue: venous blood, red: arterial blood) and norbuprenorphine (green: venous blood) predicted and observed plasma concentration-time profiles after intravenous administration of buprenorphine in adults. (**a**,**b**): selection of internal training dataset, (**c**–**f**): selection of external test dataset. Population simulations (n = 100) are shown as lines with shaded areas. Observed data are shown as circles ± standard deviation if available. References with numbers in parentheses link to a specific observed dataset described in the study table (Table 1). Predicted and observed area under the concentration-time curve from the first to the last data point (AUC_last_) and maximum plasma concentration (C_max_) values are compared in Table S6 of the Supplementary Materials. Predicted and observed plasma concentration-time profiles of all studies in adults are shown in Section 4.1 of the Supplementary Materials both on a linear and a semilogarithmic scale. iv: intravenous, m.d.: multiple dose.

The whole-body PBPK model for adults precisely predicts plasma concentration-time profiles of buprenorphine and norbuprenorphine following intravenous administration of buprenorphine. Visual comparison of predicted to observed plasma profiles are shown in Figure 2 (selection of internal and external dataset) and in detail in Section 4.1 of the Supplementary Materials (all studies, both linear and semilogarithmic plots). Predicted plasma profile trajectories are in close agreement with profiles observed both for buprenorphine venous and arterial blood plasma concentrations as well as for norbuprenorphine plasma concentrations.

**Figure 3:**
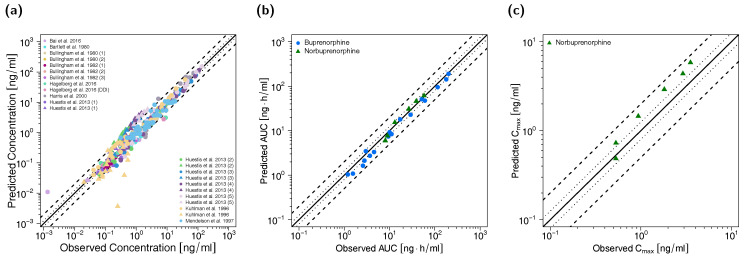
Predicted versus observed plasma concentrations (**a**) as well as predicted versus observed AUC_last_ (**b**) and C_max_ (**c**) values of buprenorphine and norbuprenorphine for the adult PBPK model. In (**a**), each symbol represents a single plasma concentration (circles: buprenorphine, triangles: norbuprenorphine). In (**b**,**c**), each symbol represents the AUC_last_ or C_max_ of a single plasma concentration-time profile (blue circles: buprenorphine, green triangles: norbuprenorphine). As stated in the materials and methods section, C_max_ values were only calculated for long-term infusions and norbuprenorphine metabolite. The black solid lines mark the lines of identity. Black dotted lines indicate 1.25-fold, black dashed lines indicate 2-fold deviation. AUC_last_: area under the plasma concentration-time curve from the first to the last data point, C_max_: maximum plasma concentration.

All predicted AUC_last_ and C_max_ values are within the 2-fold acceptance criterion. The goodness-of-fit plot of predicted versus observed plasma concentrations is shown in Figure 3 together with goodness-of-fit plots of predicted versus observed AUC_last_ and C_max_ values. The GMFE values for the adult PBPK model are 1.22 and 1.45 for AUC_last_ and C_max_, respectively. Moreover, 84% of all predicted plasma concentrations fall within 2-fold of the corresponding observed concentration. The overall MRD value for predicted plasma concentrations for the adult PBPK model is 1.70. Detailed results on MRD and GMFE values, calculated for all studies, are provided in Sections 4.4 and 4.5 of the Supplementary Materials, the results of the sensitivity analysis are shown in Section 4.6 of the Supplementary Materials.

Metabolism of buprenorphine to its major active metabolite norbuprenorphine is predominantly mediated through CYP3A4 (~65%) and CYP2C8 (~30%) [21]. In total, this pathway is responsible for about 35% of buprenorphine metabolism [21,35,52]. In contrast, urinary excretion only covers a minor fraction of buprenorphine elimination (0–1%) [26,35,62]. The PBPK model predictions for fraction metabolized to norbuprenorphine of ~37% and for fraction of buprenorphine excreted unchanged in urine of ~0.5% perfectly align with these literature reports (visual comparison of predicted to observed fractions of buprenorphine excreted unchanged in urine are shown in Figure S2 of the Supplementary Materials). Two factors, for the metabolic pathway to norbuprenorphine and the metabolic pathway to other metabolites, were estimated and multiplied with the in vitro literature values for the respective maximum reaction velocities in order to account for the in vivo relation of drug metabolized to norbuprenorphine and to other metabolites, respectively [21,35,52]. Further, the predicted fraction of the dose excreted in urine as norbuprenorphine (~2%) is in concordance with the literature as well (1.3 to 2.1%) [35]. This fraction was achieved by implementing the efflux transporter P-gp in the PBPK model according to the literature [37]. Drug-dependent parameters of the final PBPK model are depicted in Table 2. For detailed information including system-dependent model parameters, see Section 1 of the Supplementary Materials.

### 3.3. Pediatric PBPK Model Building and Evaluation

The adult PBPK model was scaled to two pediatric populations with a mean age of 5.9 years and 31 weeks (postmenstrual age), respectively. The fraction unbound of buprenorphine was calculated with the method of McNamara and Alcorn [40] and resulted in fraction unbounds of 5.1% for the child population and 7.2% (mean) for the preterm neonate population. All other drug-dependent parameters were kept fixed to the values of the adult PBPK model. Enzyme concentrations in the respective organs were scaled based on the implemented ontogeny functions [39].

**Figure 4:**
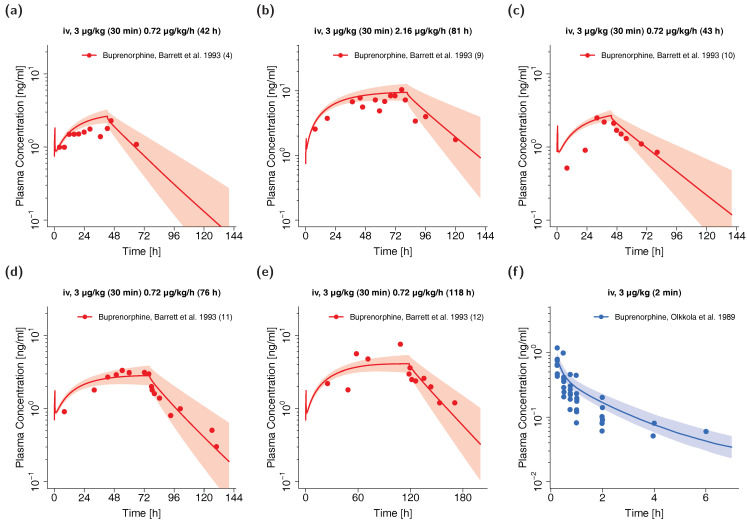
Buprenorphine (red: arterial blood, blue: venous blood) predicted and observed plasma concentration-time profiles after intravenous administration of buprenorphine in preterm neonates (**a**–**e**) and children (**f**). Population simulations (n = 100) are shown as lines with shaded areas. Observed data are shown as circles. References with numbers in parentheses link to a specific observed dataset described in Table 1 and Table S2 of the Supplementary Materials. Predicted and observed area under the plasma concentration-time curve from the first to the last data point (AUC_last_) and maximum plasma concentration (C_max_) values are compared in Table S6 of the Supplementary Materials. Predicted and observed plasma concentration-time profiles of all studies in pediatrics are shown in Section 4.2 of the Supplementary Materials both on a linear and a semilogarithmic scale. iv: intravenous.

Visual comparison of predicted to observed individual plasma profiles are shown in Figure 4 (selection of plots) and in detail in Section 4.2 of the Supplementary Materials (all plots, both linear and semilogarithmic).

**Figure 5:**
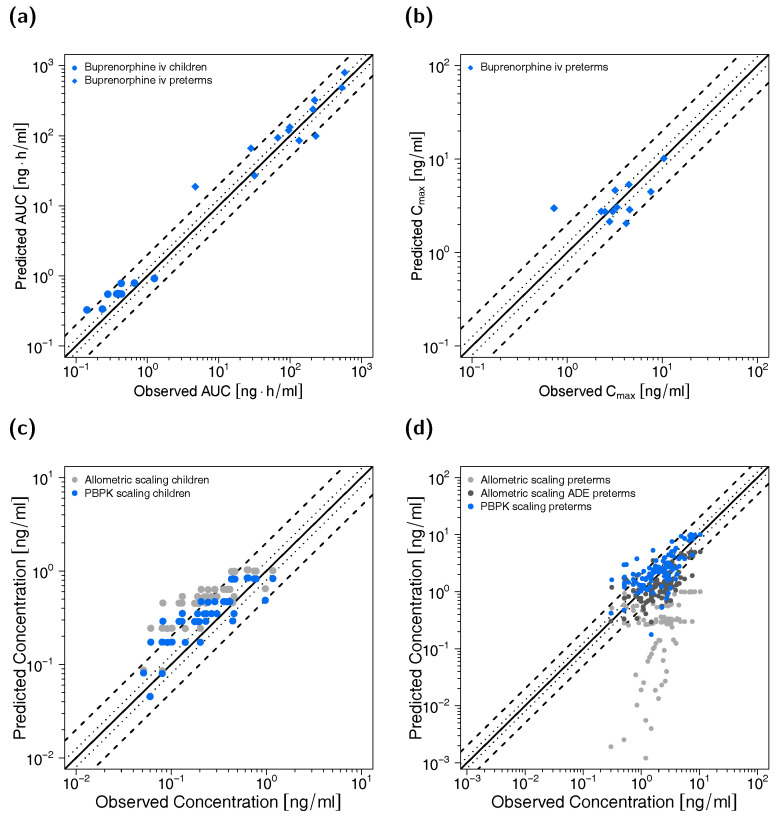
Predicted versus observed AUC_last_ (**a**) and C_max_ (**b**) values of buprenorphine for the pediatric PBPK models as well as predicted versus observed plasma concentrations for children (**c**) (blue: PBPK modeling, grey: allometric scaling) and preterm neonates (**d**) (blue: PBPK modeling, grey: allometric scaling, dark grey: allometric scaling with ADE as suggested by Mahmood and Tegenge [69]). In (**a**,**b**), each symbol represents the AUC_last_ or C_max_ of a single concentration-time profile. In (**c**,**d**), each symbol represents a single plasma concentration. As stated in the materials and methods section, C_max_ values were only calculated for long-term infusions. The black solid lines mark the lines of identity. Black dotted lines indicate 1.25-fold, black dashed lines indicate 2-fold deviation. ADE: age-dependent exponent, AUC_last_: area under the plasma concentration-time curve from the first to the last data point, C_max_: maximum plasma concentration.

Goodness-of-fit plots of predicted to observed AUC_last_ and C_max_ values are shown in Figure 5 accompanied with goodness-of-fit plots of predicted versus observed plasma concentrations. The GMFE values for individual AUC_last_ predictions were 1.54 for the child and 1.57 for the preterm neonate population, respectively. Ninety percent of individual AUC_last_ predictions for the child population and 75% of individual AUC_last_ predictions for the preterm neonate population were within 2-fold of the respective observed values. GMFE of C_max_ was 1.44 for the long-term infusions in preterm neonates (with 83% of individual C_max_ predictions within 2-fold range). Moreover, 81% (children) and 80% (preterm neonates) of all predicted plasma concentrations fell within 2-fold of the corresponding observed concentrations (overall MRD values of 1.72 for plasma concentration predictions in children and 1.86 for predictions in preterm neonates). Detailed results for MRD and GMFE values for the pediatric predictions can be found in Sections 4.4 and 4.5 of the Supplementary Materials. The allometric scaling approach led to less precise predictions (see Figure 5c,d) with MRD values of 2.28 (children), 12.46 (preterm neonates without age-dependent exponent) and 2.08 (preterm neonates with age-dependent exponent).

### 3.4. DDI Evaluation with the Adult PBPK Model

**Figure 6:**
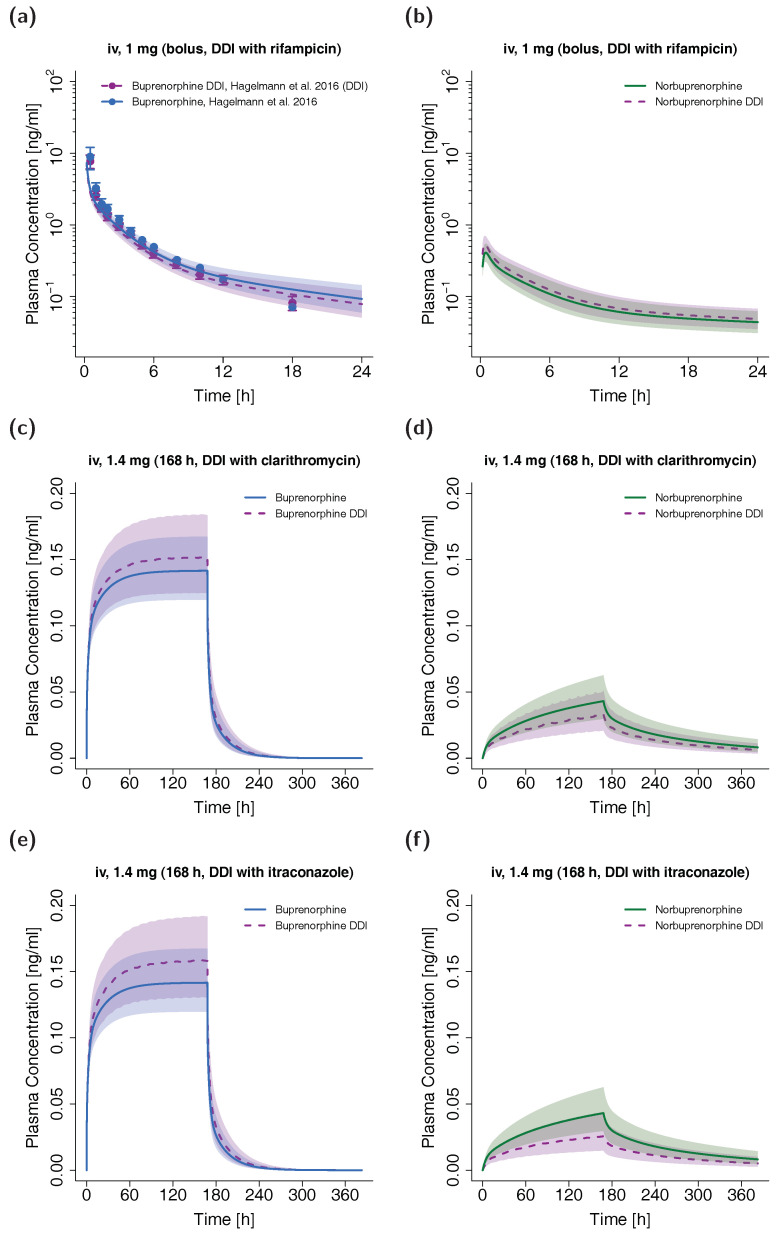
DDI scenarios for buprenorphine (blue, left panel) and norbuprenorphine (green, right panel) with the perpetrator drugs rifampicin ((**a**,**b**), semilogarithmic), clarithromycin ((**c**,**d**), linear) and itraconazole ((**e**,**f**), linear) in adults. Buprenorphine and norbuprenorphine plasma concentrations during concomitant administration of a DDI perpetrator drug are shown in purple. Population simulations (n = 100) are shown as lines with shaded areas. If available, observed data are shown as filled circles ± standard deviation (**a**). References link to a specific observed dataset described in Table 1. Information about dosing regimens as well as observed and predicted AUC ratios for buprenorphine and norbuprenorphine are depicted in Table 3. DDI, drug-drug interaction; iv, intravenous.

The plasma concentration-time profiles of the simulated DDI scenarios are depicted in Figure 6. A slight decrease in buprenorphine AUC could be observed when simulating buprenorphine administration with concomitant rifampicin compared to simulation of buprenorphine administration alone in the setting of the DDI study by Hagelberg et al. [26]. The corresponding ratio of predicted AUC_ratio_ (0.89) and observed AUC_ratio_ (0.85) for the DDI was 0.96. The predicted AUC_ratio_ for norbuprenorphine was 1.13 (see Table 3). For the assessment of DDI potential of buprenorphine with clarithromycin and itraconazole, a dosing regimen of a long-term buprenorphine infusion was selected to achieve similar steady-state plasma concentrations compared with the administration of marketed transdermal patches with 10 µg/h buprenorphine [70]. The administration of the perpetrator drugs started prior to buprenorphine administration and continued throughout the administration of the buprenorphine infusion. For details regarding the dosing regimens see Table 3. The predicted AUC_ratio_ of buprenorphine for the DDI with clarithromycin and itraconazole was 1.06 and 1.11, respectively, while the predicted AUC_ratio_ of norbuprenorphine was calculated to be 0.82 and 0.64.

## 4. Discussion

In this study, whole-body PBPK models of buprenorphine for an adult and two pediatric populations have been successfully developed. The adult PBPK model provides a consistent representation of the buprenorphine and norbuprenorphine dose–exposure relationship following intravenous administration of a wide dose range (0.3–16 mg) and describes and predicts buprenorphine and norbuprenorphine venous and arterial plasma concentration-time profiles. Thereby, predictions of the fraction of buprenorphine metabolized to norbuprenorphine and fractions of buprenorphine and norbuprenorphine excreted in urine align with literature reports. With the successful scaling of the adult PBPK model to children and preterm neonates, we confirm the potential of PBPK modeling to predict the PK in pediatrics. Moreover, we demonstrate the applicability of scaling an adult PBPK model to preterm neonates in order to predict individual plasma profiles with 75% of AUC ratios falling within 2-fold range. The performance of the PBPK models have been demonstrated by comparison of predicted to observed plasma concentration-time profiles and the respective goodness-of-fit plots, the calculation of MRD values as well as the comparison of predicted to observed AUC_last_ and C_max_ values including the calculation of the respective GMFEs.

By defining absorption, distribution, metabolism, and excretion (ADME) as a function of anatomy, physiology and biochemistry, PBPK modeling offers the opportunity of rational scaling between adults and children [31,33]. This study investigated the prediction of individual AUCs and plasma concentrations of 22 individual buprenorphine plasma profiles. In the case of predictions for children at the age of 4.6–7.5 years, 90% of individual AUC predictions were within 2-fold range. In the case of predictions for preterm neonates with 27–34 weeks of postmenstrual age, 75% of individual AUC and 83% of C_max_ predictions were within 2-fold range, suggesting good predictive model performance. While PK predictions for preterm neonates are particularly challenging [71,72,73], our results provide evidence that individual predictions of AUC and C_max_ values can be feasible.

As reported recently in other pediatric PBPK modeling approaches [12], the clearance in children (age range of 1–12 years) was slightly underestimated. This could be partly due to the fact that the implemented ontogeny functions for the CYP and UGT enzymes do not account for partially elevated concentrations in this age group, which has been reported in literature [39,74].

PK predictions with the PBPK modeling approach were superior compared to the allometric approach, especially for the preterm neonate population. The application of the exponent 1.2 for the allometric scaling of clearance in preterm neonates led to an improvement of predictions in this population compared to the exponent of 0.75, supporting the suggested advantages of an age-dependent exponent in allometric scaling by Mahmood and Tegenge [69].

In contrast to the simulated DDI scenario with rifampicin (decrease in buprenorphine AUC of ~11%), concomitant itraconazole administration slightly elevated the AUC of buprenorphine (~11%) due to the inhibition of CYP3A4. Similarly, clarithromycin inhibited the metabolism of buprenorphine to norbuprenorphine through CYP3A4 (AUC elevation of ~6%), while the CYP2C8 and UGT metabolic pathways were not affected by the DDIs with itraconazole and clarithromycin. Recent studies with the perpetrator drugs voriconazole and rifampicin have shown stronger DDI effects after oral and sublingual buprenorphine administration [24,25,26,75]. This is probably due to the fact that first-pass metabolism in the gut, which can be highly affected by DDIs, is avoided during intravenous buprenorphine administration. As a result, the DDI assessment in this study rather reflects the DDI potential for buprenorphine administrations not affected by first-pass metabolism like intravenous and transdermal applications.

Albeit clarithromycin (mechanism-based inhibition) and itraconazole (competitive inhibition) strongly inhibit CYP3A4 metabolism to norbuprenorphine, AUCs of norbuprenorphine did not vanish (decrease of only ~18% and ~36%, respectively). Firstly, norbuprenorphine can also be produced through CYP2C8. Secondly, the additional inhibition of the efflux transporter P-gp leads to a decreased norbuprenorphine excretion in the model. The simulated DDI scenario with rifampicin led to a less pronounced effect on the AUC of norbuprenorphine (increase of ~11%) despite an effect of comparable extent on buprenorphine AUC. This can be attributed to a simultaneous induction and inhibition of norbuprenorphine’s production (CYP2C8 and CYP3A4) and elimination pathways (UGT1A1 and UGT1A3) by rifampicin.

As plasma concentrations of norbuprenorphine-glucuronide were not available in the included studies, enterohepatic circulation for norbuprenorphine was not implemented in the PBPK model. To account for this missing process, a factor for maximum reaction velocities of UGT1A1 and UGT1A3 norbuprenorphine metabolism was estimated to decrease norbuprenorphine elimination. However, this could still lead to underpredictions of norbuprenorphine plasma levels, especially in terminal phases, multiple-dose regimens and DDI scenarios. Hence, the simulated DDI effects on norbuprenorphine plasma concentrations (increase with coadministration of rifampicin, decrease with coadministration of clarithromycin and itraconazole) have to be interpreted carefully. Moreover, only a limited number of PK studies with reported norbuprenorphine measurements were available for PBPK model building and evaluation [6,59]. Kapil et al. have reported “slightly higher” norbuprenorphine plasma levels after transdermal buprenorphine application during concomitant use of ketoconazole (inhibitor of CYP3A4 and P-gp), which “may be explained by ketoconazole inhibition of the efflux transporter” [3]. The concomitant administration of voriconazole, an inhibitor of CYP3A4 and CYP2C19, with oral buprenorphine led to an increase of norbuprenorphine AUC of ~400% in a recent study [24]. The authors hypothesized that the elevation of norbuprenorphine levels could be due to inhibition of transporters like P-gp among others, which could affect tissue distribution. The inhibition of P-gp did not result in such an increase of norbuprenorphine plasma concentrations in the simulated DDI scenarios. However, if implemented in the model, an enhanced enterohepatic circulation due to inhibition of P-gp might explain the observed increase. Further studies with buprenorphine need to be conducted to investigate the effect of DDIs on norbuprenorphine exposure including distribution and elimination through transport proteins.

Buprenorphine has recently been of interest in mechanistic modeling efforts. Kalluri et al. and Johnson et al. developed two intravenous and sublingual models of buprenorphine with the SimCyp^®^ simulator [53,76]. The model by Kalluri et al. represents an adult PBPK model and was further extended to a pregnant population by Zhang et al. [77]. Ji et al. used the model to assess the influence of benzodiazepines on buprenorphine PK, which was shown to be negligible [78]. While Johnson et al. succeeded in predicting clearance parameters in adults and 6-year-old children, the observed clearance in a younger age group fell “at the bottom end of the predicted results in term newborns” [76]. This could possibly be due to the fact that only CYP3A4 and UGT1A1 were incorporated in the model and considered for ontogeny. The focus of this study was on predictions of clearance values in different populations. Predictions of buprenorphine plasma concentration-time profiles in adults or pediatrics after intravenous administration were not shown.

Moreover, neither of the models included norbuprenorphine, a major active metabolite of buprenorphine [37]. The contribution of norbuprenorphine to the analgesic efficacy seen after buprenorphine administration is still under debate [79]. However, norbuprenorphine showed a higher potency with regard to the induction of dose-related respiratory depression compared to buprenorphine [80], which has recently been confirmed in a clinical trial with sublingual buprenorphine, pointing out the relevance of the metabolite norbuprenorphine [81]. All AUC_last_ and C_max_ values of norbuprenorphine plasma concentration-time profiles predicted with the presented PBPK model lie within 2-fold range of the corresponding observed values with an MRD value of 2.27 for the predicted plasma concentrations.

Norbuprenorphine plasma concentration measurements were only available in clinical studies with adults. Hence, PBPK model predictions for norbuprenorphine were only evaluated in this population. Furthermore, the DDI assessment could only be carried out with the adult PBPK model due to the fact that the incorporated perpetrator drug PBPK models were developed for the application in non-pediatric populations [22].

The impact of the inhibition processes of UGT1A1 and UGT1A3 by buprenorphine and norbuprenorphine on the AUC values was negligible as seen in the local sensitivity analysis. This is probably due to the fact that intracellular unbound drug concentrations were far below the respective K_i_ values from the literature [34], which is supported by Kress in a recent review [23]. As a result, these inhibitory processes seem to play a minor role in the fate of buprenorphine and norbuprenorphine PK if the in vitro K_i_ values can be transferred to the in vivo setting and the range of predicted intracellular concentrations reflects the in vivo scenario.

Transdermal buprenorphine has shown its benefits in the treatment of diverse acute and chronic pain syndromes as well as other difficult-to-treat pain conditions and OUD [18,82]. Sustained-release formulations such as transdermal patches hold the potential to reduce plasma concentration fluctuations and risk for non-adherence. Moreover, recent studies have evaluated the use of transdermal buprenorphine patches in children and its reduced risk of ADR compared to other dosage forms with the need for further investigations [3]. As a result of the good predictive PK performance, the new established intravenous buprenorphine PBPK models could be used to develop transdermal PBPK models for predictions of buprenorphine plasma concentrations after transdermal administration based on patch characteristics and in vitro dissolution data.

## 5. Conclusions

A whole-body parent-metabolite PBPK model of buprenorphine has been developed to predict buprenorphine and norbuprenorphine venous and arterial blood plasma concentration-time profiles as well as buprenorphine and norbuprenorphine urinary excretion after intravenous administration in adults. The model has been used for the assessment of buprenorphine DDIs with clarithromycin, itraconazole as well as rifampicin. Furthermore, the adult PBPK model has been successfully scaled to both a child and a preterm neonate population for predictions of individual plasma concentration-time profiles. The models are thoroughly documented in the Supplementary Materials and publicly available in the OSP repository. With that, the models could support the development of a physiological transdermal buprenorphine model, contribute to a library of PBPK models for predictions in other DDI scenarios, and help with future investigations of buprenorphine and norbuprenorphine pharmacokinetics, including the design of clinical trials and novel formulations both for adults and pediatrics.

## Figures and Tables

**Table 1:** Overview of clinical studies used for building and evaluation of the PBPK models.

Clinical Study	Dose [mg]	Administration	n	Female [%]	Age [Years]	Weight [kg]	Blood Sample ^a^	Norbuprenorphine Measurements	Dataset	Reference
*Adults*										
Bai et al. 2016	0.3	iv (2 min)	25	24	35.5 (20–53)	76.1 (62.6–93.0)	arterial	no	e	[54]
Bartlett et al. 1980	0.3	iv (1 min) ^b^	1	-	-	-	arterial	no	e	[55]
Bullingham et al. 1980 (1)	0.3	iv (1 min)	24	42	64.5 ± 1.6	67.7 ± 2.4	arterial	no	e	[56]
Bullingham et al. 1980 (2)	0.3	iv (1 min, m.d.)	10	40	67.5 ± 6.5	67.5 ± 2.1	arterial	no	e	[56]
Bullingham et al. 1982 (1)	0.3	iv (1 min)	5	60	66.8 ± 2.9	65.0 ± 4.0	arterial	no	e	[57]
Bullingham et al. 1982 (2)	0.3	iv (1 min)	5	60	64.2 ± 2.5	66.4 ± 2.9	arterial	no	i	[57]
Bullingham et al. 1982 (3)	0.3	iv (1 min)	5	60	66.0 ± 3.2	64.8 ± 3.9	arterial	no	e	[57]
Everhart et al. 1999	1	iv (60 min)	6	-	-	-	-	yes	i	[35]
Hagelberg et al. 2016 (1)	1	iv (bolus)	12	42	- (19–23)	- (57–95)	venous	no	e	[26]
Hagelberg et al. 2016 (2) ^c^	1	iv (bolus)	12	42	- (19–23)	- (57–95)	venous	no	e	[26]
Harris et al. 2000	4	iv (10 min)	9	11	34 (21–42)	-	venous	no	e	[58]
Huestis et al. 2013 (1)	2	iv (1 min)	5	-	34.6 (32–39)	74.7 (62.1–82.6)	venous	yes	i	[59]
Huestis et al. 2013 (2)	4	iv (1 min)	5	-	34.6 (32–39)	74.7 (62.1–82.6)	venous	yes	e	[59]
Huestis et al. 2013 (3)	8	iv (1 min)	5	-	34.6 (32–39)	74.7 (62.1–82.6)	venous	yes	e	[59]
Huestis et al. 2013 (4)	12	iv (1 min)	5	-	34.6 (32–39)	74.7 (62.1–82.6)	venous	yes	e	[59]
Huestis et al. 2013 (5)	16	iv (1 min)	5	-	34.6 (32–39)	74.7 (62.1–82.6)	venous	yes	i	[59]
Kuhlman et al. 1996	1.2	iv (1 min)	5	0	34.4 (27–40)	67.7 (62.6–72.7)	venous	yes	i	[6]
Mendelson et al. 1997	1	iv (30 min)	6	17	29 (21–38)	-	venous	no	e	[60]
*Pediatrics*										
Barrett et al. 1993 ^d^	3 µg/kg + 0.72–2.16 µg/kg/h	iv (30 min + 11–118 h)	12	-	31 weeks (27–34) ^e^	1.5 (0.9–2.4)	arterial	no	e	[51]
Olkkola et al. 1989	3 µg/kg	iv (2 min)	10	-	5.9 (4.6–7.5)	21.4 (18.5–25)	venous	no	e	[61]

-: not available, e: external test dataset, i: internal training dataset, iv: intravenous, m.d.: multiple dose, ^a^ if sample information was not specified, venous blood samples were assumed, ^b^ administration time not given; based on the observed data and information from other studies, an administration time of 1 min was assumed, ^c^ with concomitant administration of rifampicin, ^d^ detailed information on individual patient characteristics and dosing regimens is depicted in Table S2 of the Supplementary Materials, ^e^ postmenstrual age.

**Table 2:** Buprenorphine and norbuprenorphine drug-dependent parameters.

Parameter	Value	Unit	Source	Literature	Reference	Value	Unit	Source	Literature	Reference	Description
	Buprenorphine					Norbuprenorphine					
MW	467.64	g/mol	lit.	467.64	[63] ^a^	413.55	g/mol	lit.	413.55	[63] ^b^	Molecular weight
pK_a1_ (base)	12.54		lit.	12.54	[63] ^a^	10.49		lit.	10.49	[63] ^b^	Acid dissociation constant
pK_a2_ (acid)	7.50		lit.	7.50	[63] ^a^	9.80		lit.	9.80	[63] ^b^	Acid dissociation constant
logP	3.40		lit.	3.40	[64]	3.19		lit.	3.19	[65] ^c^	Lipophilicity
f_u_ (adults)	4.0	%	lit.	4.0	[66]	21.7	%	optim.	-	-	Fraction unbound
f_u_ (children)	5.1	%	calc.	5.1	[40,66]						Fraction unbound
f_u_ (preterm neonates)	7.2	%	calc.	7.2	[40,66]						Fraction unbound
CYP2C8 K_m_ -> norbup	5.2	µmol/L	lit.	5.2	[21] ^e^						Michaelis-Menten constant
CYP2C8 v_max_ -> norbup	f_1_∙176.3	pmol/min/mg protein	lit.	176.3	[21]						Maximum reaction velocity
CYP3A4 K_m_ -> norbup	5.7	µmol/L	lit.	5.7	[21] ^e^						Michaelis-Menten constant
CYP3A4 v_max_ -> norbup	f_1_∙520.0	pmol/min/mg protein	lit.	520.0	[21]						Maximum reaction velocity
CYP3A4 K_m_ -> undef	5.7	µmol/L	ass.	-	-						Michaelis-Menten constant
CYP3A4 v_max_ -> undef	f_2_∙1352.1	pmol/min/mg protein	calc.^d^	-	-						Maximum reaction velocity
CYP3A7 K_m_ -> undef	29.1	µmol/L	calc.^d^	-	-						Michaelis-Menten constant
CYP3A7 v_max_ -> undef	f_2_∙632.6	pmol/min/mg protein	calc.^d^	-	-						Maximum reaction velocity
UGT1A1 K_m_ -> undef	10.4	µmol/L	lit.	10.4	[34] ^e^	21.8	µmol/L	lit.	21.8	[34] ^e^	Michaelis-Menten constant
UGT1A1 v_max_ -> undef	f_2_∙6726.8	pmol/min/mg protein	lit.	6726.8	[34]	f_3_∙714.6	pmol/min/mg protein	lit.	714.6	[34]	Maximum reaction velocity
UGT1A3 K_m_ -> undef	1.1	µmol/L	lit.	1.1	[34] ^e^	14.7	µmol/L	lit.	14.7	[34] ^e^	Michaelis-Menten constant
UGT1A3 v_max_ -> undef	f_2_∙642.6	pmol/min/mg protein	lit.	642.6	[34]	f_3_∙387.0	pmol/min/mg protein	lit.	387.0	[34]	Maximum reaction velocity
UGT2B7 K_m_ -> undef	1.8	µmol/L	lit.	1.8	[34] ^e^						Michaelis-Menten constant
UGT2B7 v_max_ -> undef	f_2_∙823.8	pmol/min/mg protein	lit.	823.8	[34]						Maximum reaction velocity
P-gp K_m_						3.4	µmol/L	optim.	-		Michaelis-Menten constant
P-gp k_cat_						2.14	1/min	optim.	-		Transport rate constant
f_1_	2.80		optim.	-							Factor
f_2_	0.27		optim.	-							Factor
f_3_						0.43		optim.	-		Factor
GFR fraction	1.00		ass.	-		1.00		ass.	-		Filtered drug in the urine
UGT1A1 K_i_	14.8	µmol/L	lit.	14.8	[34] ^e^						Conc. for 50% inhibition
UGT1A3 K_i_	0.5	µmol/L	lit.	0.5	[34] ^e^	1.6	µmol/L	lit.	1.6	[34] ^e^	Conc. for 50% inhibition
Partition coefficients	Diverse		calc.	Schmitt	[67]	Diverse		calc.	PK-Sim	[38]	Cell to plasma partitioning
Cellular permeability	6.91E-03	cm/min	calc.	PK-Sim	[38]	8.91E-03	cm/min	calc.	PK-Sim	[38]	Perm. into the cellular space

-: not available, ass.: assumed, calc.: calculated, conc.: concentration, CYP: cytochrome P450, GFR: glomerular filtration rate, lit.: literature, norbup: norbuprenorphine, optim.: optimized, perm.: permeability, P-gp: P-glycoprotein, PK-Sim: PK-Sim standard calculation method, Schmitt: Schmitt calculation method, undef: undefined metabolite, UGT: uridine 5ʹ-diphospho-glucuronosyltransferase, ^a^ DrugBank entry for buprenorphine. https://www.drugbank.ca/drugs/DB00921, accessed 21 April 2020, ^b^ DrugBank entry for norbuprenorphine. https://www.drugbank.ca/metabolites/DBMET00174, accessed 21 April 2020, ^c^ HMDB entry for norbuprenorphine. https://hmdb.ca/metabolites/HMDB0060546, accessed 21 April 2020, ^d^ for detailed information please refer to Section 1 in the Supplementary Materials; ^e^ apparent K_i_ and K_m_ literature values were corrected according to [68] using the free fractions of buprenorphine and norbuprenorphine in microsome assays from [36].

**Table 3:** DDI study dosing regimens with predicted and observed AUC ratios.

Victim Drug [Dose]	Perpetrator Drug [Dose]	Pred. AUC Ratio (Buprenorphine) with/without Perpetrator	Obs. AUC Ratio (Buprenorphine) with/without Perpetrator	Pred. AUC Ratio (Norbuprenorphine) with/without Perpetrator	Obs. AUC Ratio (Norbuprenorphine) with/without Perpetrator	Reference
Buprenorphine [1.4 mg, 168 h iv infusion]	Clarithromycin [250 mg, bid, po]	1.06	-	0.82	-	simulated ^a^
Buprenorphine [1.4 mg, 168 h iv infusion]	Itraconazole [100 mg, bid, po] ^b^	1.11	-	0.64	-	simulated ^a^
Buprenorphine [1 mg, iv bolus]	Rifampicin [600 mg, qd, po]	0.89	0.85	1.11	-	[26]

-: not available, bid: twice daily, iv: intravenous, obs.: observed, po: oral, pred.: predicted, qd: once daily, ^a^ population simulations were carried out with a virtual population (n = 100) with an age range of 20–50 years and without specific body weight or height restrictions as implemented in PK-Sim^®^, ^b^ loading dose of 200 mg as first dose.

## Supplementary Materials

The following are available online at https://www.mdpi.com/1999-4923/12/6/578/s1, Electronic Supplementary Materials: Additional detailed model information and evaluation.
