# Supplementary material for: Physiologically-Based Pharmacokinetic (PBPK) Modeling of Buprenorphine in Adults, Children and Preterm Neonates
Source: Pharmaceutics. 2020 Jun 23;12(6):578. doi: 10.3390/pharmaceutics12060578 (PMC7355427; doi:10.3390/pharmaceutics12060578)
Supplement: Supplementary file 1 [file pharmaceutics-12-00578-s001.pdf]

Pharmaceutics

Supplementary Materials: Physiologically-Based Pharmacokinetic (PBPK) Modeling of Buprenorphine in Adults, Children and Preterm Neonates

Lukas Kovar, Christina Schräpel, Dominik Selzer, Yvonne Kohl, Robert Bals, Matthias Schwab and Thorsten Lehr

Contents

1 PBPK Model Building **2**
1.1 PBPK Model Building – General 2
1.2 System-dependent Parameters and Virtual Populations 3

2 Drug-Drug-Interaction (DDI) Modeling **6**
2.1 DDI Modeling – General 6
2.2 Mathematical Implementation of DDIs 7
2.2.1 Competitive Inhibition 7
2.2.2 Mechanism-Based Inhibition (MBI) 7
2.2.3 Induction 8

3 Allometric Scaling **9**

4 PBPK Model Evaluation **11**
4.1 Adult PBPK Model Evaluation 12
4.2 Pediatric PBPK Model Evaluation 20
4.3 Quantitative PBPK Model Evaluation 26
4.4 Mean Relative Deviation (MRD) Values of Buprenorphine and Norbuprenorphine Plasma Concentration Predictions 26
4.5 Geometric Mean Fold Error (GMFE) of AUC_{last} and C_{max} Predictions 28
4.6 Buprenorphine and Norbuprenorphine PBPK Model Sensitivity Analysis 30

References **32**

1 PBPK Model Building

1.1 PBPK Model Building – General

In agreement with pediatric physiologically based pharmacokinetic (PBPK) model development workflows, first, an adult PBPK model was built and evaluated with observed plasma profiles to gain confidence in the parametrization of the PBPK model, before the model was scaled to pediatric populations [1–4]. The general model building process is described in the methods section of the main manuscript. This includes the implementation of important distribution and elimination processes including cytochrome P450 (CYP) and uridine 5'-diphospho-glucuronosyltransferase (UGT) enzymes as well as transporters. For the buprenorphine model these are the metabolism of buprenorphine to norbuprenorphine through CYP3A4 and CYP2C8 [5], the metabolism pathways metabolizing buprenorphine to other non-specified metabolites through CYP3A4, CYP3A7, UGT1A1, UGT1A3 and UGT2B7 as well as renal excretion through glomerular filtration.

For the metabolite norbuprenorphine metabolism through UGT1A1 and UGT1A3 as well as renal clearance by glomerular filtration and tubular secretion through the transport protein P-glycoprotein (P-gp) were implemented in the model [6, 7] . The respective Michaelis-Menten constants (K_m) and maximum reaction velocities (v_{max}) were obtained from published *in vitro* experiments [5, 8]. As nonspecific binding influences K_m and K_i values in *in vitro* assays in microsomes, the values need to be adjusted by multiplication with fraction unbound in the microsomal assay ($f_{u,mic}$) [9]. Hence, the obtained literature values of K_m and K_i were multiplied by measured $f_{u,mic}$ values of buprenorphine (0.42) and norbuprenorphine (0.84), respecitvely [6]. The enzyme CYP3A4 catalyzes two different metabolic pathways of buprenorphine, the metabolism to norbuprenorphine (R_1) and a reaction, in which norbuprenorphine is not the product substance (R_2) [5]. For the latter one, no specific K_m and v_{max} values were reported. Hence, the $K_{m,\ R_2}$ value was assumed to be the same as for R_1 and $v_{max,\ R_2}$ was calculated to be a multiple of $v_{max,\ R_1}$ using the amount of buprenorphine consumed and the amount of norbuprenorphine produced, respectively, from the *in vitro* study by Picard et al. yielding a $v_{max,\ R_2}$ value of 1352.1 pmol/min/mg microsomal protein [5].

Studies have shown that CYP3A7 is involved in buprenorphine metabolism [5, 10]. CYP3A7 is the major fetal form of CYP3A [11]. Hence, CYP3A7 can be important for PK predictions of CYP3A substrates in pediatrics and therefore was incorporated in our model for predictions in pediatrics. K_m and v_{max} values for the metabolism of buprenorphine through CYP3A7 have not been reported. However, a study by Williams et al. provides information on the relative metabolic capabilities of CYP3A4 and CYP3A7 to metabolize a structurally diverse set of molecules (n=15) by comparing K_m [µmol/L] and v_{max} [nmol/min/nmol P450] values [11]. The dataset was extended by three more molecules including their respective K_m and v_{max} values from a recently published study [12]. On average, K_m values for CYP3A7 were 5.1 times higher compared to the respective K_m values of CYP3A4 for the model substances, v_{max} values were 75% lower. These factors were used and multiplied with the K_m and v_{max} values for the metabolism of buprenorphine through CYP3A4 (5.7 µmol/L and 12.5 pmol/min/pmol P450, calculated from 1352.1 pmol/min/mg microsomal protein and the content of CYP3A4 enzyme of 108 pmol P450/mg microsomal protein in liver microsomes [5, 13, 14]) to obtain the values for CYP3A7. This yields a K_m value of 29.1 µmol/L and a v_{max} value of 3.17 pmol/min/pmol P450 or 632.6 pmol/min/mg microsomal protein using the protein content of CYP3A7 enzyme of 199.57 pmol P450/mg microsomal protein in fetal liver microsomes [15].

According to the literature, about 35% of buprenorphine is metabolized to norbuprenorphine [5, 16, 17]. In order to achieve this amount, two factors for the metabolism to norbuprenorphine and the metabolism to other metabolites, respectively, were estimated and multiplied with the *in vitro* literature values for the respective maximum reaction velocities (see Table 2 in the main manuscript).

1.2 System-dependent Parameters and Virtual Populations

PBPK modeling enables mechanistic representation of drug disposition in virtual individuals. Virtual individuals with all system-dependent physiological parameters such as blood flow rates and organ compositions were generated in PK-Sim® based on the demographic characteristics of the respective study population (see Table 1 in the manuscript and Table S2). The applied algorithms for the generation of virtual individuals have been previously reported [18]. If no information on study demographics was available, a standard 30-year-old male was assumed with weight and height values according to the PK-Sim® database.

Virtual populations of 100 individuals for each study were set up according to the population demographics of each respective simulated study. If no age range was specified, virtual populations were created with individuals 20 to 50 years of age and without specific body weight or height restrictions as implemented in PK-Sim®. In the generated virtual populations, demographics such as age, height, weight and corresponding organ volumes, tissue compositions, blood flow rates, etc. were varied by an implemented algorithm in PK-Sim® within the limits of the ICRP (International Commission on Radiological Protection) or NHANES (National Health and Nutrition Examination Survey) databases [19, 20]. Tissue expression distributions of the enzymes and proteins were provided in the PK-Sim® expression database according to the literature [21–23].

Additionally, variability of the expression levels of the implemented drug metabolizing enzymes CYP2C8, CYP3A4, CYP3A7, UGT1A1, UGT1A3 and UGT2B7 as well as of the transport protein P-gp was implemented. System-dependent parameters, such as information on reference concentrations and the respective variabilities of metabolizing enzymes and transporters are shown in Table S1. Population predictions were plotted as geometric mean with geometric standard deviation. If all individual concentration-time datasets were available but demographic values could not be matched to the specific profile, median with 90% population prediction intervals were plotted.

Table S1: System-dependent parameters and expression of relevant enzymes and transporters.

Enzyme / Transporter	Mean reference concentration [µmol/L][a]	Geometric standard deviation of the reference concentration in adults[b]	Relative expression in the different organs[c]	Ontogeny function	Half-life liver [hours]	Half-life Intestine [hours]
**Enzymes**						
CYP2C8	2.56 [14]	2.05 [24]	RT-PCR [21]	[24]	23	23
CYP3A4	4.32 [14]	1.18 (liver)[24]<br>1.45 (intestine)[24]	RT-PCR [21]	[24]	36	23
CYP3A7	7.98 [15]	1.25 [24]	RT-PCR [21]	[24]	36	23
UGT1A1	1.30 [25]	1.37 [24]	RT-PCR [23]	[24]	36	23
UGT1A3	0.40 [25]	1.60[d]	RT-PCR [23]	[24][d]	36	23
UGT2B7	2.78 [25]	1.60 [24]	EST [26]	[24]	36	23
**Transporters**						
P-gp	1.41 [27]	1.60 [28]	RT-PCR [22][e]	-	36	23

CYP: cytochrome P450, **EST**: Expressed Sequence Tags, **P-gp**: P-glycoprotein, **RT-PCR**: reverse transcription polymerase chain reaction, **UGT**: uridine 5'-diphospho-glucuronosyltransferase

[a] [µmol protein/L] in the tissue of the highest expression

[b] for information on geometric standard deviation in pediatrics, please refer to [24]

[c] PK-Sim® expression database profile

[d] since no specific ontogeny function for UGT1A3 is implemented in PK-Sim®, the same ontogeny function as for UGT2B7 was assumed based on ontogeny information in [29]

[e] with the relative expression in intestinal mucosa increased by factor 3.57 according to [27]

Table S2: Extension of Table 1 in the main manuscript with detailed information on the demographics and dosing regimens of the study by Barrett et al. [30]

**Clinical study**	**Loading dose**[a] (30 min) [µg/kg]	**Second Dose**[a] [µg/kg/h]	**Infusion Time (second dose) [h]**	**n**	**Female [%]**	**Age**[b] **[weeks]**	**Weight [kg]**	**Blood sample**	**Norbuprenorphine measurements**
Barrett et al. 1993 (1)	3.00	0.72	48	1	-	31	1.5	arterial	no
Barrett et al. 1993 (2)	3.00	0.72	24	1	-	30	0.9	arterial	no
Barrett et al. 1993 (3)	3.00	0.72	11	1	-	32	1.3	arterial	no
Barrett et al. 1993 (4)	3.00	0.72	42	1	-	31	1.8	arterial	no
Barrett et al. 1993 (5)	3.00	0.72	42	1	-	30	1.5	arterial	no
Barrett et al. 1993 (6)	3.00	1.44	23	1	-	28	1.2	arterial	no
Barrett et al. 1993 (7)	3.00	1.44	77	1	-	31	1.1	arterial	no
Barrett et al. 1993 (8)	3.00	0.72	42	1	-	34	1.8	arterial	no
Barrett et al. 1993 (9)	3.00	2.16	81	1	-	30	1.6	arterial	no
Barrett et al. 1993 (10)	3.00	0.72	43	1	-	32	2.4	arterial	no
Barrett et al. 1993 (11)	3.00	0.72	76	1	-	31	1.6	arterial	no
Barrett et al. 1993 (12)	3.00	0.72	118	1	-	27	1.0	arterial	no

-: not available

[a] intravenous administration

[b] postmenstrual age

2 Drug-Drug-Interaction (DDI) Modeling

2.1 DDI Modeling – General

Rifampicin is both an inhibitor and inducer of different CYP enzymes. This includes the enzymes CYP2C8, CYP3A4, UGT1A1 and UGT1A3 as well as the transporter P-gp among others [31–39]. A previously developed rifampicin PBPK model [27] was used for the DDI assessment and was extended by interaction constants describing the induction of CYP2C8, UGT1A1 and UGT1A3 as well as the competitive inhibition of CYP2C8, UGT1A1 and UGT1A3 by rifampicin. The parameters of the extended rifampicin model are shown in Table S3.

Table S3: Drug-dependent parameters of the rifampicin PBPK model (adopted from [27])

Parameter	Value	Unit	Source	Literature	Reference	Description
MW	822.94	g/mol	Literature	822.94	[40]	Molecular weight
pKa (acid)	1.70	-	Literature	1.70	[41]	First acid dissociation constant
pKa (base)	7.90	-	Literature	7.90	[41]	Second acid dissociation constant
Solubility (pH 7.5)	2.80	g/l	Literature	2.80	[42]	Solubility
logP	2.50	-	Optimized	1.30, 2.70	[40, 43]	Lipophilicity
f_u	17.00	%	Literature	17.00	[36]	Fraction unbound
B/P ratio	0.89	-	Calculated	0.90[a]	[44]	Blood/plasma ratio
OATP1B1 K_m	1.50	µmol/l	Literature	1.50	[45]	OATP1B1 Michaelis-Menten constant
OATP1B1 k_{cat}	105.41	1/min	Optimized	-	-	OATP1B1 transport rate constant
AADAC K_m	195.10	µmol/l	Literature	195.10	[46]	AADAC Michaelis-Menten constant
AADAC k_{cat}	9.87	1/min	Optimized	-	-	AADAC catalytic rate constant
P-gp K_m	55.00	µmol/l	Literature	55.00	[47]	P-gp Michaelis-Menten constant
P-gp k_{cat}	0.61	1/min	Optimized	-	-	P-gp transport rate constant
GFR fraction	1.00	-	Assumed	-	-	Fraction of filtered drug in the urine
EHC continuous fraction	1.00	-	Assumed	-	-	Fraction of bile continually released
Induction EC_{50}	0.34	µmol/l	Literature	0.80*0.42	[36, 48]	Conc. for half-maximal induction
E_{max} OATP1B1	0.38	-	Optimized	-	-	Maximum *in vivo* induction effect
E_{max} OATP1B3	0.38	-	Assumed	-	-	Maximum *in vivo* induction effect
E_{max} AADAC	0.99	-	Optimized	-	-	Maximum *in vivo* induction effect
E_{max} P-gp	2.50	-	Literature	2.50	[38]	Maximum *in vivo* induction effect
E_{max} CYP2C8	3.20	-	Literature	3.20	[39]	Maximum *in vivo* induction effect
E_{max} CYP3A4	9.00	-	Literature	9.00	[36]	Maximum *in vivo* induction effect
E_{max} UGT1A1	1.30	-	Literature	1.30	[34]	Maximum *in vivo* induction effect
E_{max} UGT1A3	1.40	-	Literature	1.40	[35]	Maximum *in vivo* induction effect
OATP1B1 K_i	0.48	µmol/l	Literature	0.48	[49]	Conc. for half-maximal inhibition
OATP1B3 K_i	0.90	µmol/l	Literature	0.90	[50]	Conc. for half-maximal inhibition
P-gp K_i	169.00	µmol/l	Literature	169.00	[37]	Conc. for half-maximal inhibition
CYP2C8 K_i	30.20	µmol/l	Literature	30.20	[31]	Conc. for half-maximal inhibition
CYP3A4 K_i	18.50	µmol/l	Literature	18.50	[31]	Conc. for half-maximal inhibition
UGT1A1 K_i	33.00	µmol/l	Literature	33.00	[33]	Conc. for half-maximal inhibition
UGT1A3 K_i	600.00	µmol/l	Literature	600.00	[32]	Conc. for half-maximal inhibition
Partition coefficients	Diverse	-	Calculated	R&R	[51, 52]	Cell to plasma partition coefficients
Cellular permeability	2.93E-05	cm/min	Calculated	PK-Sim	[13]	Permeability into the cellular space
Intestinal permeability	1.24E-05	cm/min	Optimized	3.84E-07	Calculated	Transcellular intestinal permeability
Formulation	Solution					

AADAC: arylacetamide deacetylase, **conc**: concentration, **CYP**: cytochrome P450, **EHC**: enterohepatic circulation, **GFR**: glomerular filtration rate, **OATP**: organic anion transporting polypeptide, **P-gp**: P-glycoprotein, **PK-Sim**: PK-Sim standard calculation method, **R&R**: Rodgers and Rowland calculation method, **UGT**: uridine 5'-diphospho-glucuronosyltransferase

[a] Blood/serum concentration ratio

For the simulation of the DDI with itraconazole and clarithromycin two previously published PBPK models were used [27]. The parameters of both models can be found in the supplementary material of the respective publication [27].

The DDI simulations presented in the manuscript depict pure predictions. No DDI study was used for model input parameter estimation during buprenorphine and norbuprenorphine PBPK model

development. Interaction parameters necessary for DDI simulation were obtained from literature or from the published DDI perpetrator PBPK models. With that, the adult PBPK model could not only be evaluated by its predictive performance with the test dataset but also by prediction of a DDI study [53].

2.2 Mathematical Implementation of DDIs

2.2.1 Competitive Inhibition

Competitive inhibition describes the reversible binding of an inhibitor to the active site of an enzyme or transporter and hence, the competition of substrate and inhibitor for binding. This inhibition process can be overcome by high substrate concentrations leading to a concentration-dependency. As a result of competitive inhibition v_{max} is not affected, while K_m is increased through the inhibition yielding $K_{m,app}$ (Equation S1). The reaction velocity (v) for the substrate during concomitant administration with a competitive inhibitor is described by Equation S2 [13]:

$$K_{m,app} = K_m \cdot \left(1 + \frac{[I]}{K_i}\right) \tag{S1}$$

$$v = \frac{v_{max} \cdot [S]}{K_{m,app} + [S]} \tag{S2}$$

with $K_{m,app}$ = Michaelis-Menten constant in the presence of inhibitor, K_m = Michaelis-Menten constant, $[I]$ = free inhibitor concentration, K_i = dissociation constant of the inhibitor-enzyme/transporter complex, v = reaction velocity, v_{max} = maximum reaction velocity, $[S]$ = free substrate concentration.

2.2.2 Mechanism-Based Inhibition (MBI)

Mechanism-based inhibition (MBI) is an irreversible type of inhibition. *De novo* synthesis of the inactivated protein and clearance of the mechanism-based inactivator is required to return to baseline activity of the enzyme or transporter (time-dependency). In the case of MBI, the protein degradation rate constant (k_{deg}) is increased ($k_{deg,app}$, Equation S3), while the synthesis (R_{syn}) is not affected by the inhibition process. The protein turnover during MBI is described by Equation S4. As mechanism-based inactivators are also competitive inhibitors, the K_m in the Michaelis-Menten reaction velocity equation is substituted by $K_{m,app}$ as in Equation S5 [13]:

$$k_{deg,app} = k_{deg} + \left(\frac{k_{inact} \cdot [I]}{K_I + [I]}\right) \tag{S3}$$

$$\frac{dE(t)}{dt} = R_{syn} - k_{deg,app} \cdot E(t) \tag{S4}$$

$$v = \frac{v_{max} \cdot [S]}{K_{m,app} + [S]} = \frac{k_{cat} \cdot E(t) \cdot [S]}{K_{m,app} + [S]} \tag{S5}$$

with $k_{deg,app}$ = enzyme or transporter degradation rate constant in the presence of mechanism-based inactivator, k_{deg} = enzyme or transporter degradation rate constant, k_{inact} = maximum inactivation rate constant, $[I]$ = free inactivator concentration, K_I = concentration for half-maximal inactivation, $E(t)$ = enzyme or transporter concentration, R_{syn} = rate of enzyme or transporter synthesis, v =

reaction velocity, v_{max} = maximum reaction velocity, $[S]$ = free substrate concentration, $K_{m,app}$ = Michaelis-Menten constant in the presence of inactivator, k_{cat} = catalytic rate constant.

2.2.3 Induction

Induction of an enzyme or transporter is often mediated through activation of the transcription factor pregnane X receptor (PXR). Similarly as in the case of an MBI, the return to baseline activity requires the clearance of the inducer and degradation of the induced protein (time-dependency). However, in contrast to the MBI, in this case R_{syn} is increased ($R_{syn,app}$, Equation S6), while k_{deg} remains unchanged. The protein turnover during induction is described by Equation S7 [13]:

$$R_{syn,app} = R_{syn} \cdot \left(1 + \frac{E_{max} \cdot [Ind]}{EC_{50} + [Ind]}\right) \tag{S6}$$

$$\frac{dE(t)}{dt} = R_{syn,app} - k_{deg} \cdot E(t) \tag{S7}$$

$$v = \frac{v_{max} \cdot [S]}{K_m + [S]} = \frac{k_{cat} \cdot E(t) \cdot [S]}{K_m + [S]} \tag{S8}$$

with $R_{syn,app}$ = rate of enzyme or transporter synthesis in the presence of inducer, R_{syn} = rate of enzyme or transporter synthesis, E_{max} = maximal induction effect *in vivo*, $[Ind]$ = free inducer concentration, EC_{50} = concentration for half-maximal induction *in vivo*, $E(t)$ = enzyme or transporter concentration, k_{deg} = enzyme or transporter degradation rate constant, v = reaction velocity, v_{max} = maximum reaction velocity, $[S]$ = free substrate concentration, K_m = Michaelis-Menten constant, k_{cat} = catalytic rate constant.

3 Allometric Scaling

After the development of the adult PBPK model, the model was scaled to a children and preterm neonate population for *a priori* predictions of the PK in pediatrics as described in the methods section of the main manuscript. In order to compare the PBPK model predictions for plasma concentration-time profiles observed in pediatric patients, a classical allometric scaling approach as described by Tod et al. was used [54]. Here, the parameters of classical compartmental models are scaled by allometry from adults to the pediatric populations with:

$$CL_{pediatrics} = CL_{adults} \cdot \left(\frac{BW_{pediatrics}}{BW_{adults}} \right)^{0.75} \quad \text{(S9)}$$

$$Q_{2,\,pediatrics} = Q_{2,\,adults} \cdot \left(\frac{BW_{pediatrics}}{BW_{adults}} \right)^{0.75} \quad \text{(S10)}$$

$$Q_{3,\,pediatrics} = Q_{3,\,adults} \cdot \left(\frac{BW_{pediatrics}}{BW_{adults}} \right)^{0.75} \quad \text{(S11)}$$

$$V_{c,\,pediatrics} = V_{c,\,adults} \cdot \left(\frac{BW_{pediatrics}}{BW_{adults}} \right) \quad \text{(S12)}$$

$$V_{2,\,pediatrics} = V_{2,\,adults} \cdot \left(\frac{BW_{pediatrics}}{BW_{adults}} \right) \quad \text{(S13)}$$

$$V_{3,\,pediatrics} = V_{3,\,adults} \cdot \left(\frac{BW_{pediatrics}}{BW_{adults}} \right) \quad \text{(S14)}$$

To obtain the relevant parameters of the elimination clearance, intercompartmental clearances and volume of distributions in adults (CL, Q_2, Q_3, V_c, V_2 and V_3 of a classical three compartment model, which best described the observed plasma concentration-time profiles), the parameters were estimated with NONMEM® using the internal dataset from the PBPK modeling approach. Body weight values of the adult (71 kg) and pediatric patients (see Table 1 in the main manuscript and Table S2) were extracted from the corresponding study information. In the case of scaling the elimination clearance for preterm neonates ($CL_{\text{preterm neonates}}$), the calculation was performed both with an exponent of 0.75 and with the age-dependent exponent of 1.2 as suggested by Mahmood and Tegenge [55]:

$$CL_{preterm\ neonates,\,ADE} = CL_{adults} \cdot \left(\frac{BW_{pediatrics}}{BW_{adults}} \right)^{1.2} \quad \text{(S15)}$$

The plasma concentrations were then simulated with the scaled parameters (Table S4) and compared with the corresponding plasma concentrations observed.

Table S4: Parameters calculated with the allometric scaling approach

Clinical study	CL [ml/min] [a]	CL [ml/min] [b]	Q_2 [ml/min]	Q_3 [ml/min]	V_c [L]	V_2 [L]	V_3 [L]
Adults (internal dataset)	982.0	982.0	2980.0	554.0	29.6	105.0	676.0
Barrett et al. 1993 (1)	54.5	9.6	165.0	31.0	0.6	2.2	14.3
Barrett et al. 1993 (2)	37.8	5.3	115.0	21.0	0.4	1.4	8.8
Barrett et al. 1993 (3)	50.1	8.4	152.0	28.0	0.6	2.0	12.8
Barrett et al. 1993 (4)	61.4	12.0	186.0	35.0	0.7	2.6	16.8
Barrett et al. 1993 (5)	54.5	9.6	165.0	31.0	0.6	2.2	14.3
Barrett et al. 1993 (6)	44.9	7.1	136.0	25.0	0.5	1.7	11.1
Barrett et al. 1993 (7)	44.4	6.9	135.0	25.0	0.5	1.7	10.9
Barrett et al. 1993 (8)	61.4	11.6	186.0	35.0	0.7	2.6	16.8
Barrett et al. 1993 (9)	56.9	10.3	173.0	32.0	0.7	2.4	15.2
Barrett et al. 1993 (10)	77.5	16.9	235.0	44.0	1.0	3.6	22.9
Barrett et al. 1993 (11)	56.7	10.2	172.0	32.0	0.7	2.3	15.1
Barrett et al. 1993 (12)	41.4	6.2	126.0	23.0	0.4	1.5	9.9
Olkkola et al. 1989	400.0	400.0	1214.0	226.0	8.9	32.0	204.0

CL: elimination clearance, $\mathbf{Q_2}$: intercompartmental clearance between compartment 2 and the central compartment, $\mathbf{Q_3}$: intercompartmental clearance between compartment 3 and the central compartment, $\mathbf{V_c}$: volume of the central compartment, $\mathbf{V_2}$ **and** $\mathbf{V_3}$: peripheral compartment volumes

[a] elimination clearance parameter calculated using the allometric scaling approach without an age-dependent exponent

[b] elimination clearance parameter calculated using the allometric scaling approach with an age-dependent exponent as suggested by Mahmood and Tegenge [55]

4 PBPK Model Evaluation

The descriptive (internal training dataset) and predictive (external test dataset) performance of the PBPK model is comprehensively demonstrated in this section: Linear and semilogarithmic plots of population predictions of plasma concentration-time profiles are compared to the observed profiles for both adult and pediatric PBPK models in Figures S1, S2, S5 and S6. Further, linear plots of population predictions of fractions of buprenorphine excreted unchanged in urine as well as fraction of dose excreted in urine as norbuprenorphine are compared to measured values in Figure S2. Moreover, goodness-of-fit plots comparing predicted to observed plasma concentrations are shown in Figures S3 and S7. Predicted compared to observed area under the plasma concentration-time curves from the first to the last data point (AUC_{last}) and maximum concentrations (C_{max}) values for long-term infusions in preterm neonates and norbuprenorphine metabolite are shown in Figures S4 and S8. The mean relative deviation (MRD) values as well as the predicted and observed AUC_{last} and C_{max} values including the geometric mean fold errors (GMFE) are listed in Tables S5 and S6. A local sensitivity analysis was performed in a steady-state scenario (1.4 mg (adults), 0.7 mg (children), 0.009 mg (preterm neonates), 168 hours long-term infusion, mimicking steady-state plasma concentrations of about 0.13 ng/ml, which were achieved with an administration of marketed transdermal buprenorphine patches [56]). A detailed description and the results of the sensitivity analysis can be found in Section 4.6.

4.1 Adult PBPK Model Evaluation

In this section, linear and semilogarithmic plots of plasma concentration-time profiles, linear plots of fractions of buprenorphine dose excreted unchanged in urine and fraction of dose excreted in urine as norbuprenorphine (Figures S1 and S2), a goodness-of-fit plot of predicted compared to observed plasma concentrations (Figure S3) and goodness-of-fit plots of predicted compared to observed AUC_{last} and C_{max} values (Figure S4) after intravenous administration of buprenorphine in adults are shown.

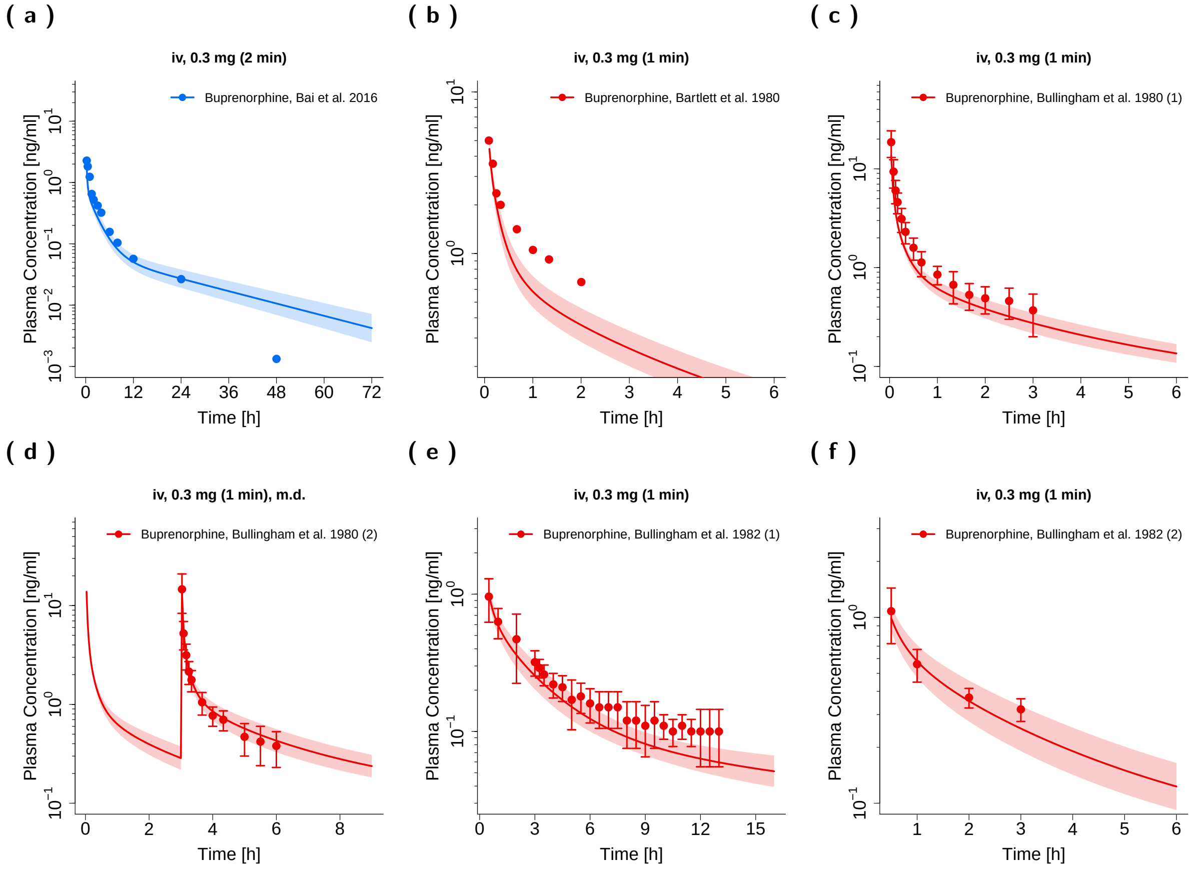

Figure S1: Buprenorphine (blue: venous blood, red: arterial blood) and norbuprenorphine (green: venous blood) plasma concentration-time profiles (semilogarithmic) after intravenous administration of buprenorphine in adults. Observed data are shown as circles, if available ± standard deviation (SD). Population simulation (n=100) geometric means are shown as lines; the shaded areas represent the predicted population geometric SD except for subfigure (q) where the line represents the population median and the shaded area the 90% population prediction interval. References with numbers in parentheses link to a specific observed dataset described in the study table (Table 1 in the main manuscript). Predicted and observed AUC_{last} and C_{max} values are compared in Table S6. **DDI**, drug-drug-interaction; **iv**, intravenous; **m.d.**, multiple dose.

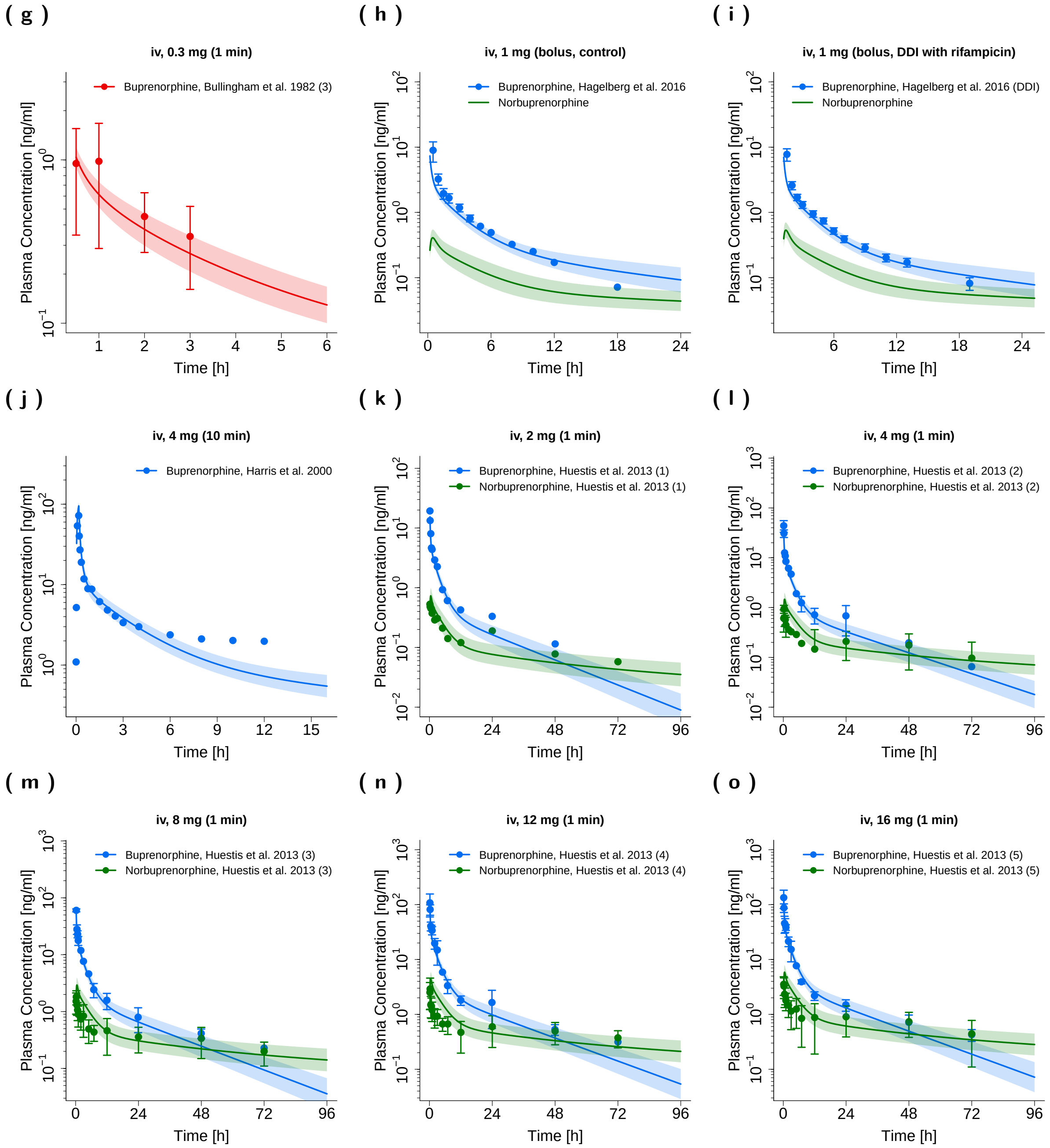

Figure S1: Buprenorphine (blue: venous blood, red: arterial blood) and norbuprenorphine (green: venous blood) plasma concentration-time profiles (semilogarithmic) after intravenous administration of buprenorphine in adults. Observed data are shown as circles, if available ± standard deviation (SD). Population simulation (n=100) geometric means are shown as lines; the shaded areas represent the predicted population geometric SD except for subfigure (q) where the line represents the population median and the shaded area the 90% population prediction interval. References with numbers in parentheses link to a specific observed dataset described in the study table (Table 1 in the main manuscript). Predicted and observed AUC_{last} and C_{max} values are compared in Table S6. **DDI**, drug-drug-interaction; **iv**, intravenous; **m.d.**, multiple dose. (continued)

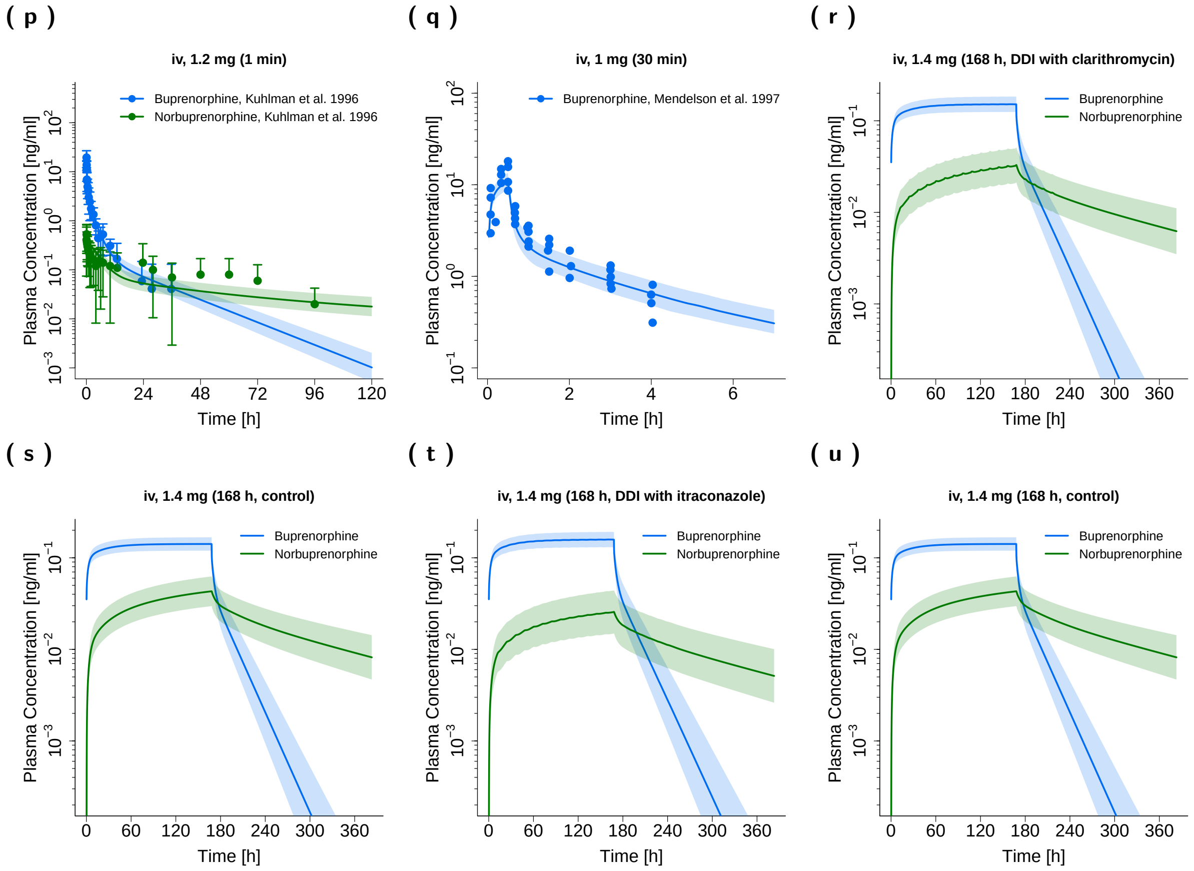

Figure S1: Buprenorphine (blue: venous blood, red: arterial blood) and norbuprenorphine (green: venous blood) plasma concentration-time profiles (semilogarithmic) after intravenous administration of buprenorphine in adults. Observed data are shown as circles, if available ± standard deviation (SD). Population simulation (n=100) geometric means are shown as lines; the shaded areas represent the predicted population geometric SD except for subfigure (q) where the line represents the population median and the shaded area the 90% population prediction interval. References with numbers in parentheses link to a specific observed dataset described in the study table (Table 1 in the main manuscript). Predicted and observed AUC_{last} and C_{max} values are compared in Table S6. **DDI**, drug-drug-interaction; **iv**, intravenous; **m.d.**, multiple dose. (continued)

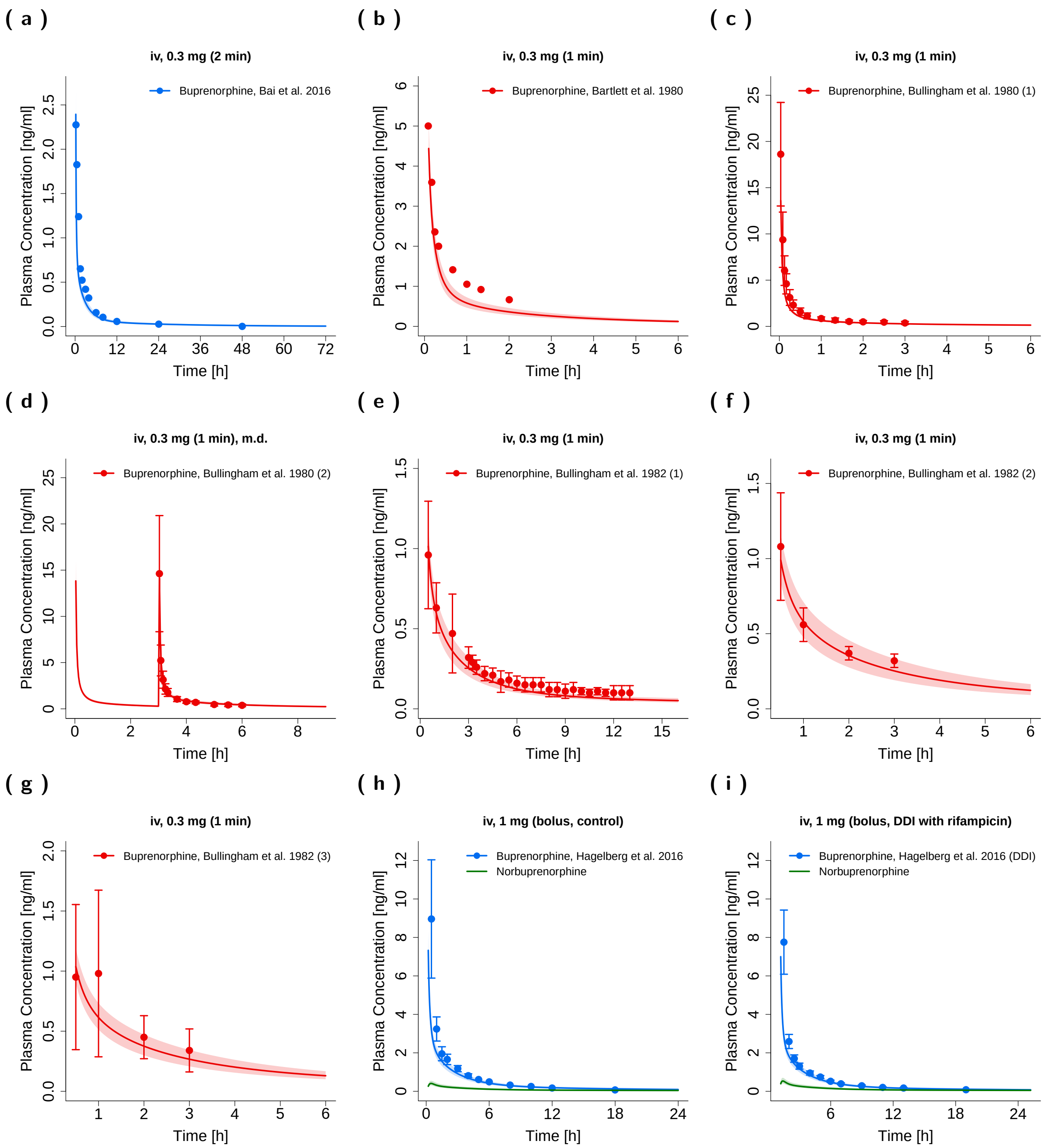

Figure S2: Buprenorphine (blue: venous blood, red: arterial blood) and norbuprenorphine (green: venous blood) plasma concentration-time profiles (linear) as well as fraction of buprenorphine (yellow) and norbuprenorphine (orange) excreted in urine after intravenous administration of buprenorphine in adults. Observed data are shown as circles, if available ± standard deviation (SD). Population simulation (n=100) geometric means are shown as lines; the shaded areas represent the predicted population geometric SD except for subfigure (q) where the line represents the population median and the shaded area the 90% population prediction interval. References with numbers in parentheses link to a specific observed dataset described in the study table (Table 1 in the main manuscript). Predicted and observed AUC_{last} and C_{max} values are compared in Table S6. **DDI**, drug-drug-interaction; **iv**, intravenous; **m.d.**, multiple dose.

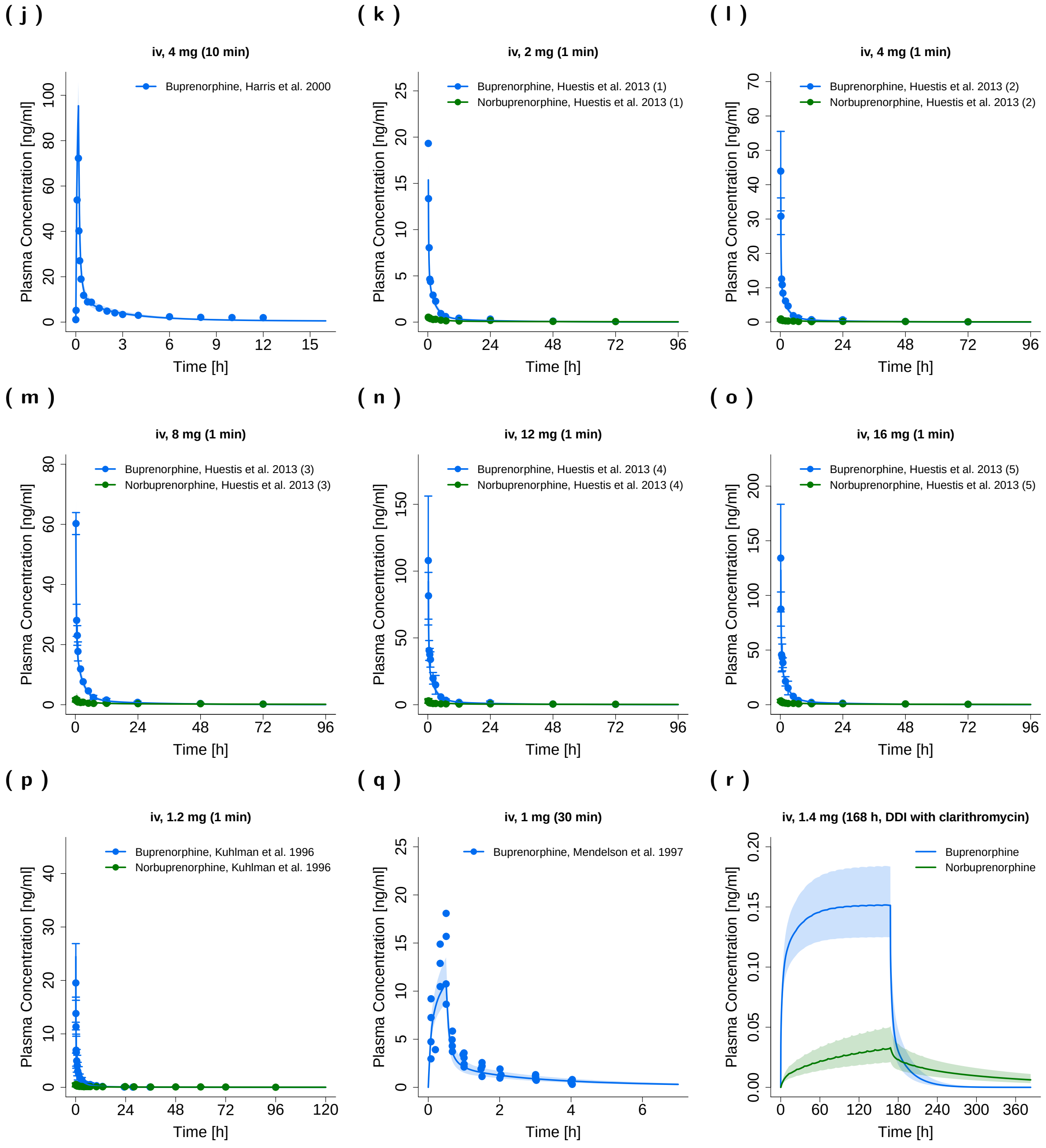

Figure S2: Buprenorphine (blue: venous blood, red: arterial blood) and norbuprenorphine (green: venous blood) plasma concentration-time profiles (linear) as well as fraction of buprenorphine (yellow) and norbuprenorphine (orange) excreted in urine after intravenous administration of buprenorphine in adults. Observed data are shown as circles, if available ± standard deviation (SD). Population simulation (n=100) geometric means are shown as lines; the shaded areas represent the predicted population geometric SD except for subfigure (q) where the line represents the population median and the shaded area the 90% population prediction interval. References with numbers in parentheses link to a specific observed dataset described in the study table (Table 1 in the main manuscript). Predicted and observed AUC_{last} and C_{max} values are compared in Table S6. **DDI**, drug-drug-interaction; **iv**, intravenous; **m.d.**, multiple dose. (continued)

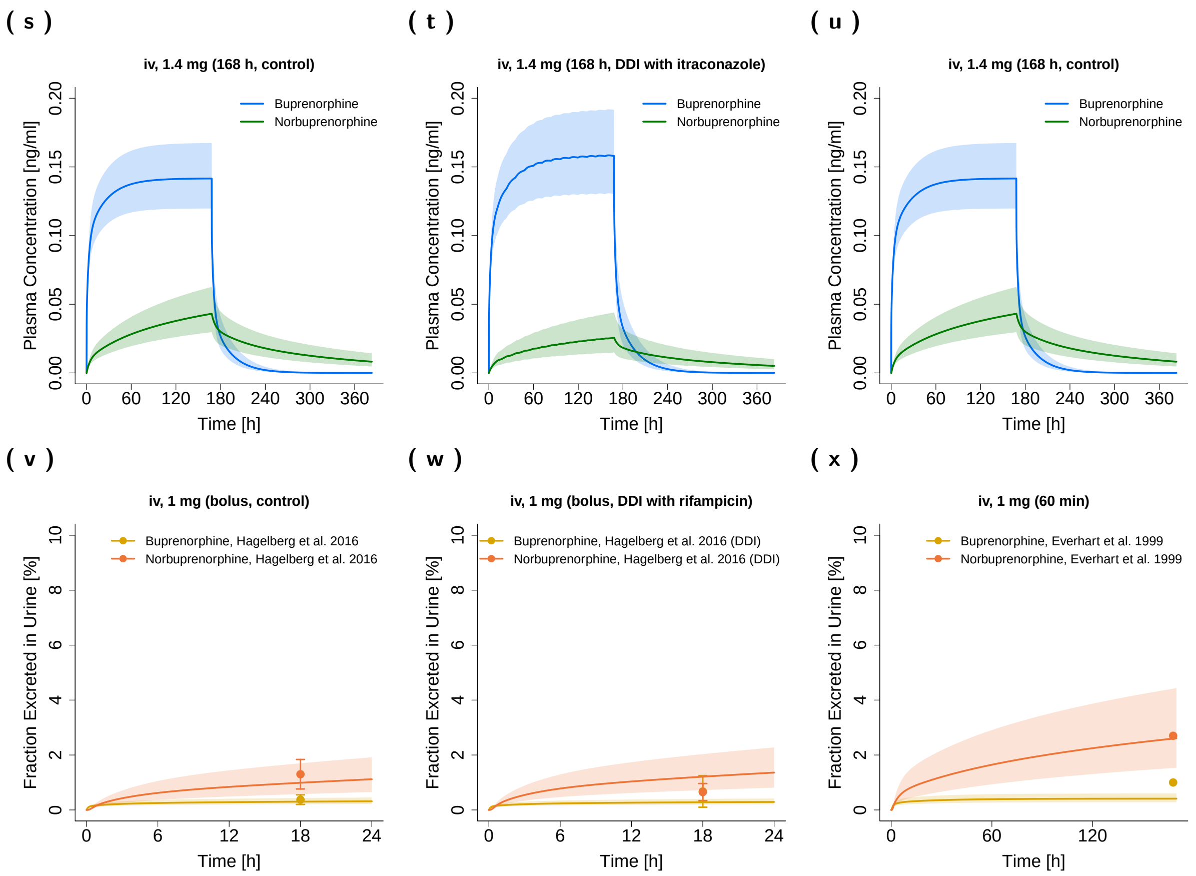

Figure S2: Buprenorphine (blue: venous blood, red: arterial blood) and norbuprenorphine (green: venous blood) plasma concentration-time profiles (linear) as well as fraction of buprenorphine (yellow) and norbuprenorphine (orange) excreted in urine after intravenous administration of buprenorphine in adults. Observed data are shown as circles, if available ± standard deviation (SD). Population simulation (n=100) geometric means are shown as lines; the shaded areas represent the predicted population geometric SD except for subfigure (q) where the line represents the population median and the shaded area the 90% population prediction interval. References with numbers in parentheses link to a specific observed dataset described in the study table (Table 1 in the main manuscript). Predicted and observed AUC_{last} and C_{max} values are compared in Table S6. **DDI**, drug-drug-interaction; **iv**, intravenous; **m.d.**, multiple dose. (continued)

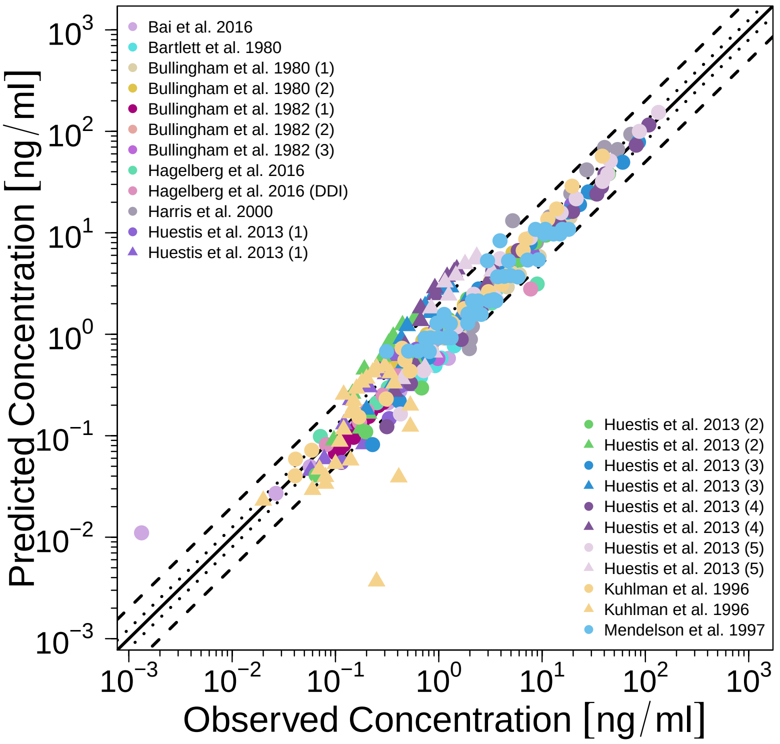

Figure S3: Predicted versus observed plasma concentrations of buprenorphine and norbuprenorphine after intravenous administration of buprenorphine in adults. The black solid line marks the line of identity. Black dotted lines indicate 1.25-fold, black dashed lines indicate 2-fold deviation.

(a) AUC

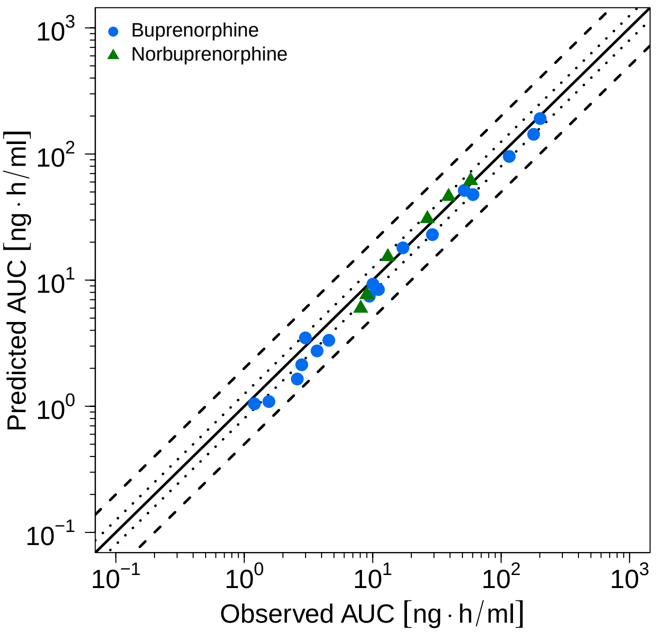

(b) C_{max}

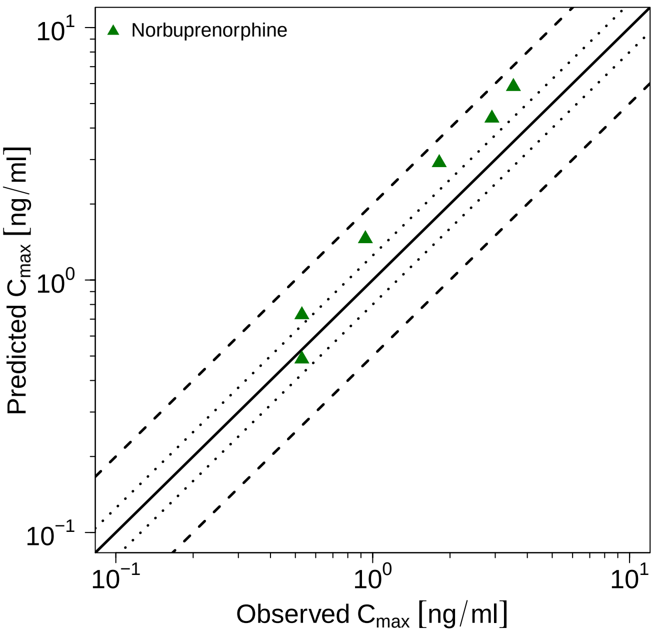

Figure S4: Predicted versus observed buprenorphine and norbuprenorphine AUC (a) and norbuprenorphine C_{max} (b) values after intravenous administration of buprenorphine in adults. C_{max} values were only calculated for long-term infusions and norbuprenorphine metabolite. Each symbol represents the AUC_{last} or C_{max} of a different plasma profile. The black solid lines mark the lines of identity. Black dotted lines indicate 1.25-fold, black dashed lines indicate 2-fold deviation. **AUC**, area under the plasma concentration-time curve from the first to the last data point; **C_{max}**, maximum plasma concentration.

4.2 Pediatric PBPK Model Evaluation

In this section, linear and semilogarithmic plots of plasma concentration-time profiles (Figures S5 and S6), goodness-of fit plots of predicted compared to observed plasma concentrations including the results of the allometric scaling approach (Figure S7) and goodness-of-fit plots of predicted compared to observed AUC_{last} and C_{max} values (Figure S8) after intravenous administration of buprenorphine in pediatrics are shown.

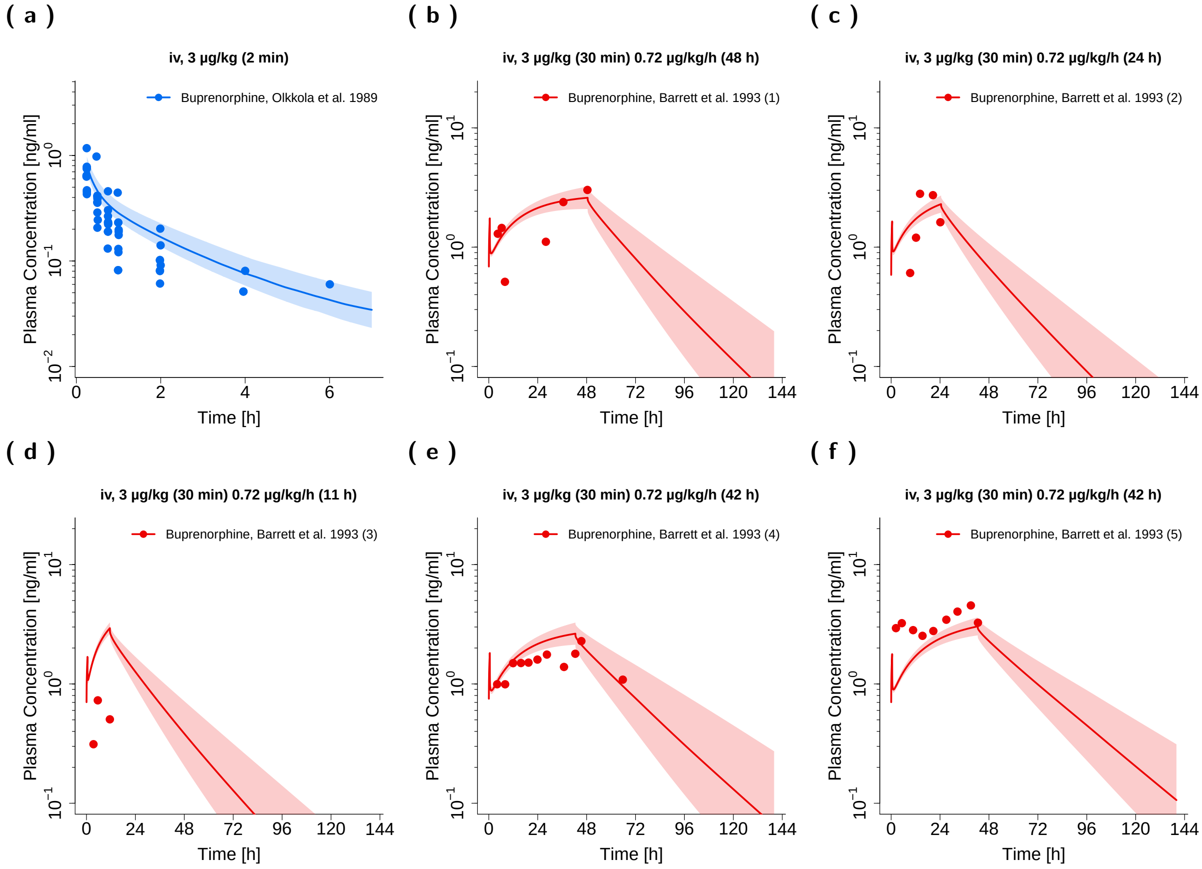

Figure S5: Buprenorphine (blue: venous blood, red: arterial blood) plasma concentration-time profiles (semilogarithmic) after intravenous administration of buprenorphine in pediatrics. Observed data are shown as circles. Population simulation (n=100) geometric means are shown as lines; the shaded areas represent the predicted population geometric SD except for subfigure (a) where the line represents the population median and the shaded area the 90% population prediction interval. References with numbers in parentheses link to a specific observed dataset described in the study table (Table 1 in the main manuscript and Table S2). Predicted and observed AUC_{last} and C_{max} values are compared in Table S6. **iv**, intravenous.

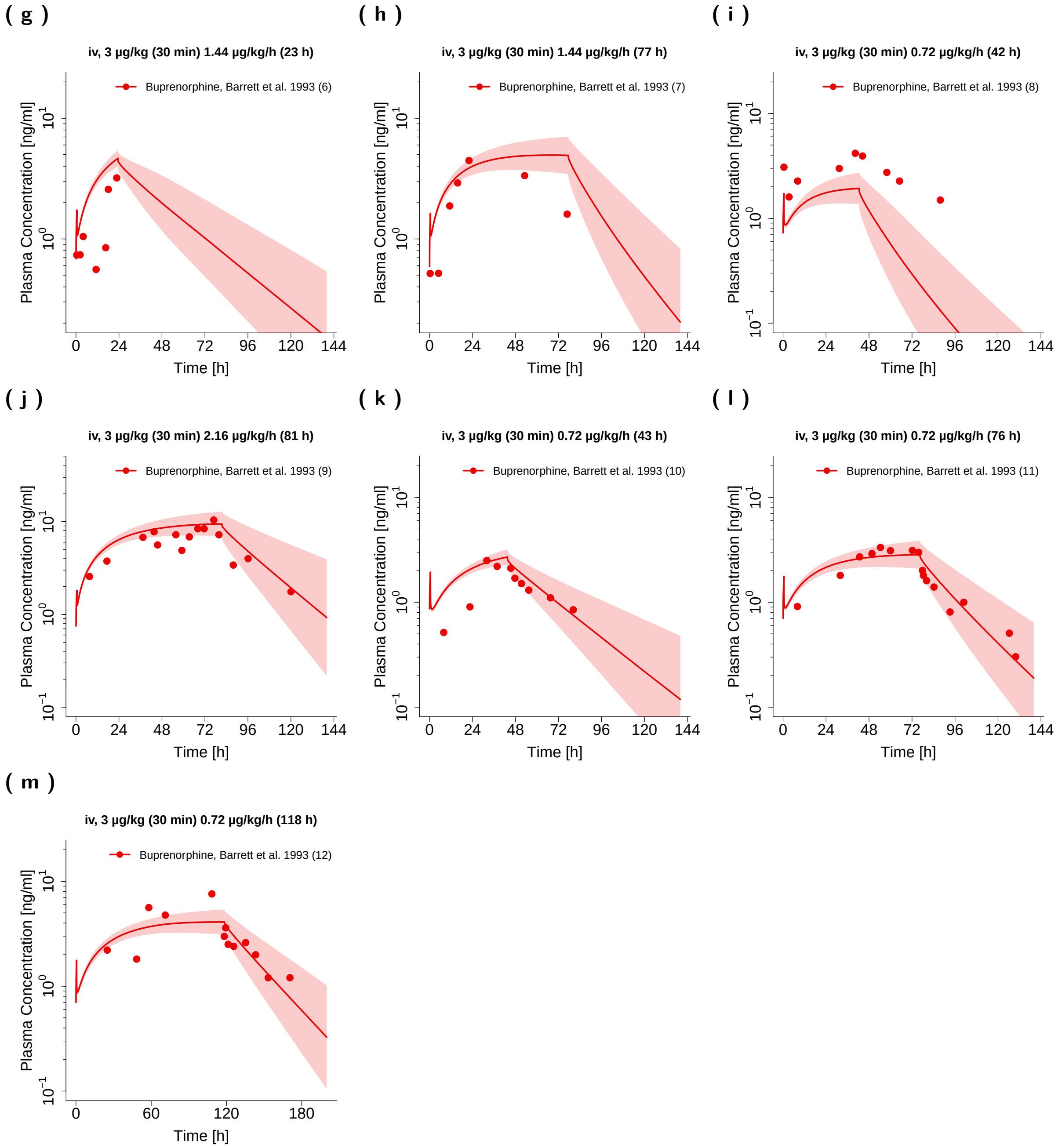

Figure S5: Buprenorphine (blue: venous blood, red: arterial blood) plasma concentration-time profiles (semilogarithmic) after intravenous administration of buprenorphine in pediatrics. Observed data are shown as circles. Population simulation (n=100) geometric means are shown as lines; the shaded areas represent the predicted population geometric SD except for subfigure (a) where the line represents the population median and the shaded area the 90% population prediction interval. References with numbers in parentheses link to a specific observed dataset described in the study table (Table 1 in the main manuscript and Table S2). Predicted and observed AUC_{last} and C_{max} values are compared in Table S6. **iv**, intravenous. (continued)

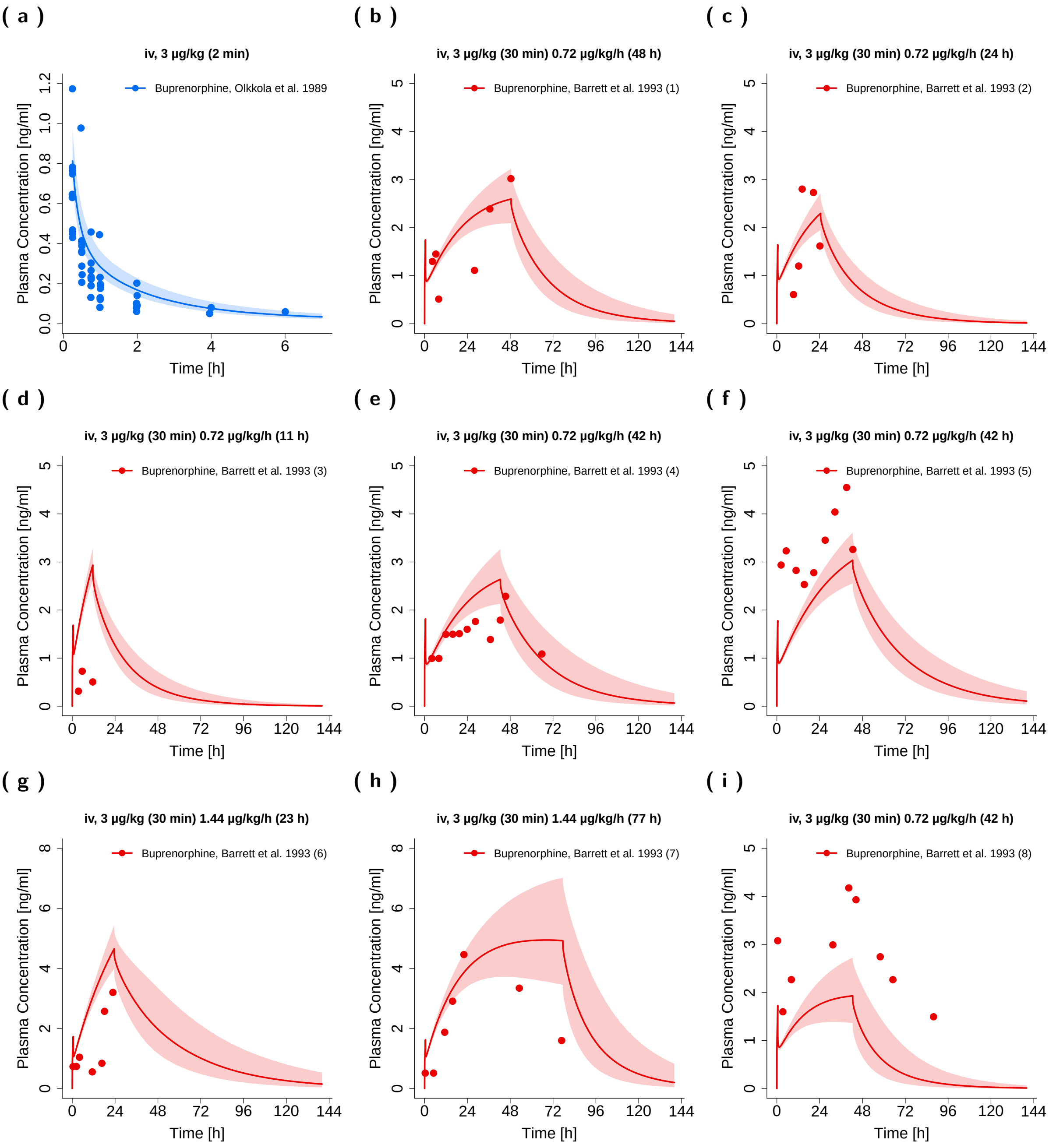

Figure S6: Buprenorphine (blue: venous blood, red: arterial blood) plasma concentration-time profiles (linear) after intravenous administration of buprenorphine in pediatrics. Observed data are shown as circles. Population simulation (n=100) geometric means are shown as lines; the shaded areas represent the predicted population geometric SD except for subfigure (a) where the line represents the population median and the shaded area the 90% population prediction interval. References with numbers in parentheses link to a specific observed dataset described in the study table (Table 1 in the main manuscript and Table S2). Predicted and observed AUC_{last} and C_{max} values are compared in Table S6. **iv**, intravenous.

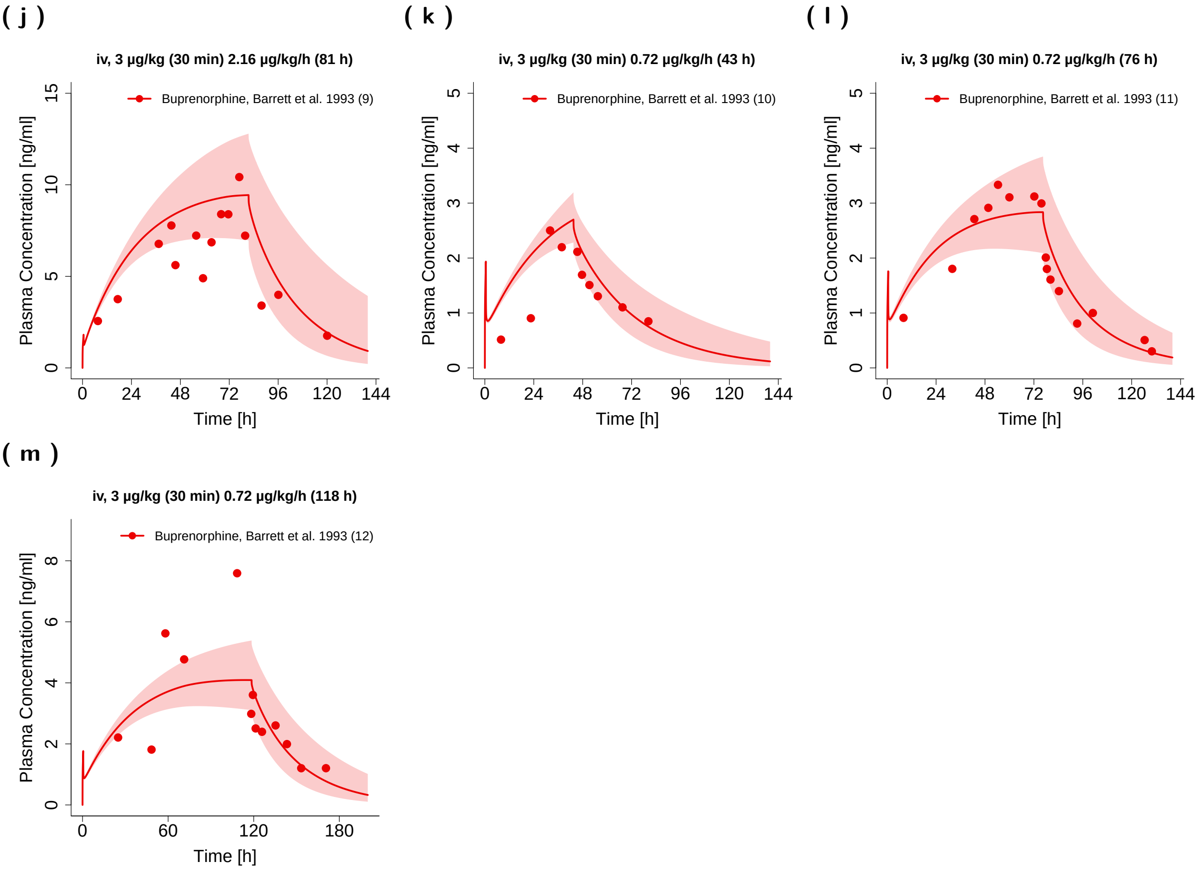

Figure S6: Buprenorphine (blue: venous blood, red: arterial blood) plasma concentration-time profiles (linear) after intravenous administration of buprenorphine in pediatrics. Observed data are shown as circles. Population simulation (n=100) geometric means are shown as lines; the shaded areas represent the predicted population geometric SD except for subfigure (a) where the line represents the population median and the shaded area the 90% population prediction interval. References with numbers in parentheses link to a specific observed dataset described in the study table (Table 1 in the main manuscript and Table S2). Predicted and observed AUC_{last} and C_{max} values are compared in Table S6. **iv**, intravenous. (continued)

(a) Children

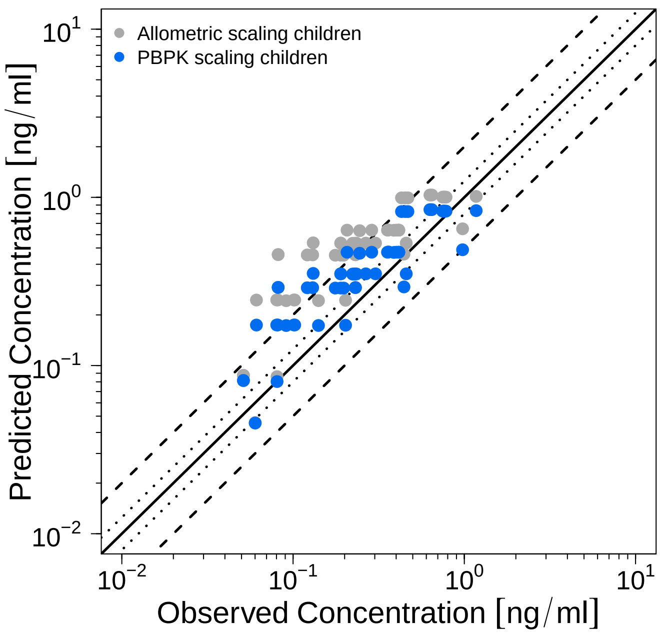

(b) Preterm neonates

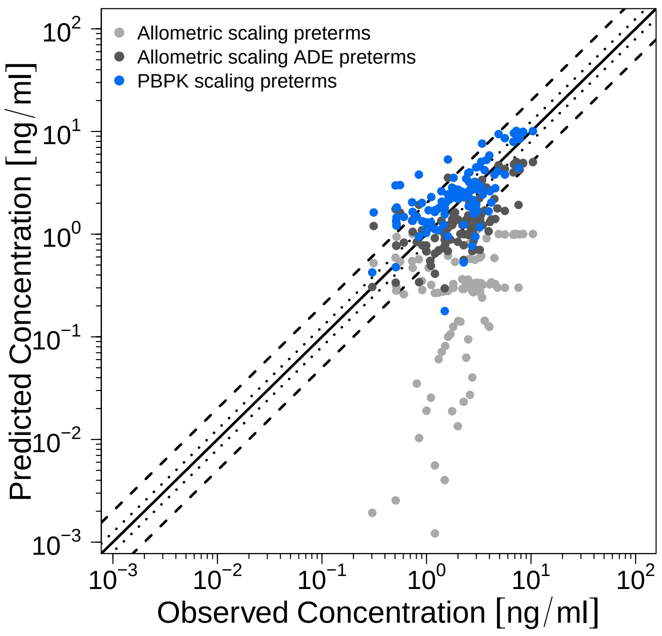

Figure S7: Predicted versus observed plasma concentrations of buprenorphine and norbuprenorphine after intravenous administration of buprenorphine in (a) children and (b) preterm neonates. Blue circles represent predicted versus observed plasma concentrations derived from the PBPK scaling approach. Light grey circles represent predicted versus observed plasma concentrations derived from the classical allometric scaling approach; dark grey circles represent predicted versus observed plasma concentrations derived from allometric scaling with an age-dependent exponent of 1.2 for preterm neonates as suggested by Mahmood and Tegenge [55] (for detailed information on the allometric scaling approach see Section 3). The black solid lines mark the lines of identity. Black dotted lines indicate 1.25-fold, black dashed lines indicate 2-fold deviation.

(a) AUC

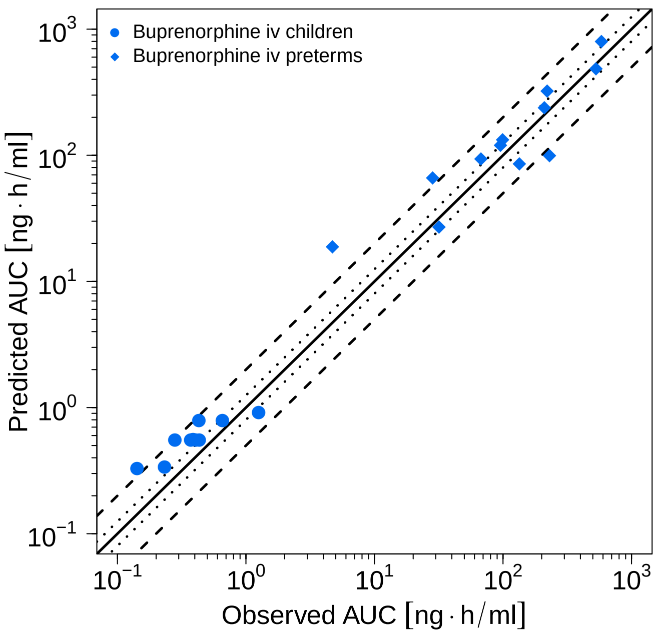

(b) C_{max}

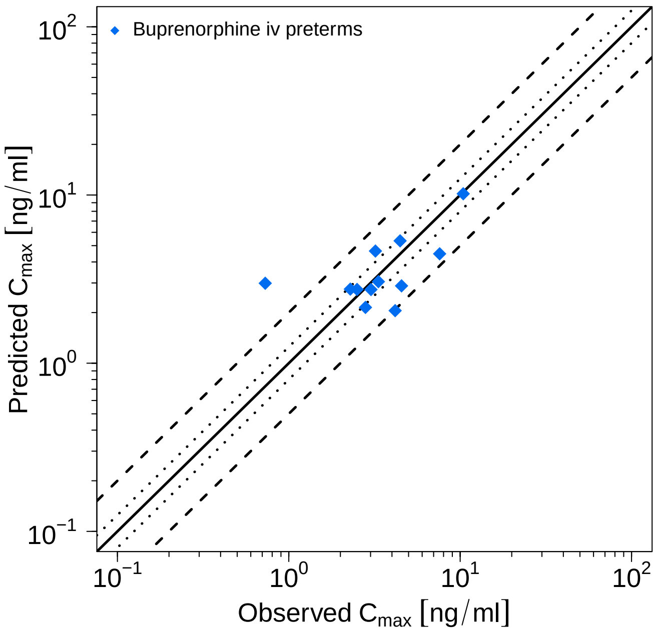

Figure S8: Predicted versus observed buprenorphine and norbuprenorphine AUC (a) and C_{max} (b) values after intravenous administration of buprenorphine in pediatrics. C_{max} values were only calculated for long-term infusions. Each symbol represents the AUC_{last} or C_{max} of a different plasma profile. The black solid lines mark the lines of identity. Black dotted lines indicate 1.25-fold, black dashed lines indicate 2-fold deviation. **AUC**, area under the plasma concentration-time curve from the first to the last data point; $\mathbf{C_{max}}$, maximum plasma concentration.

4.3 Quantitative PBPK Model Evaluation

As quantitative performance measures, mean relative deviations (MRD) of the predicted plasma concentrations for all observed and the respective predicted plasma concentrations as well as the geometric mean fold errors (GMFE) of the predicted versus observed AUC_{last} and C_{max} values were calculated according to Equation S16 and Equation S17, respectively. C_{max} values were only calculated for long-term infusions and norbuprenorphine metabolite since C_{max} values of a substance administered as intravenous bolus injection or as short-term infusions are very sensitive to the timing of blood sampling.

$$MRD = 10^x \text{ with } x = \sqrt{\frac{1}{n}\sum_{i=1}^{n}(\log_{10}\hat{c}_i - \log_{10}c_i)^2} \tag{S16}$$

Here, c_i is the ith observed plasma concentration, $\hat{c}_i$ is the respective predicted plasma concentration and n equals the number of observed values. Overall MRD values of ≤ 2 were considered as reasonable predictions [57]. MRD values for all studies are given in Table S5.

$$GMFE = 10^x \text{ with } x = \frac{1}{n}\sum_{i=1}^{n}|\log_{10}(\frac{\hat{a}_i}{a_i})| \tag{S17}$$

Here, a_i is the ith observed AUC_{last} or C_{max} value, respectively, $\hat{a}_i$ is the predicted AUC_{last} or C_{max} value, respectively, and n equals the number of studies. The calculated GMFE values are shown in Table S6.

4.4 Mean Relative Deviation (MRD) Values of Buprenorphine and Norbuprenorphine Plasma Concentration Predictions

Table S5: Mean relative deviation (MRD) values of buprenorphine and norbuprenorphine plasma concentration predictions.

Route & Dose	Compound	MRD	Reference
**Buprenorphine iv adults**			
iv, 0.3 mg (2 min)	Buprenorphine	2.06	Bai et al. 2016 [58]
iv, 0.3 mg (1 min)	Buprenorphine	1.58	Bartlett et al. 1980 [59]
iv, 0.3 mg (1 min)	Buprenorphine	1.35	Bullingham et al. 1980 (1) [60]
iv, 0.3 mg (1 min), m.d.	Buprenorphine	1.26	Bullingham et al. 1980 (2) [60]
iv, 0.3 mg (1 min)	Buprenorphine	1.37	Bullingham et al. 1982 (1) [61]
iv, 0.3 mg (1 min)	Buprenorphine	1.21	Bullingham et al. 1982 (2) [61]
iv, 0.3 mg (1 min)	Buprenorphine	1.42	Bullingham et al. 1982 (3) [61]
iv, 1 mg (bolus)	Buprenorphine	1.44	Hagelberg et al. 2016 [53]
iv, 1 mg (bolus, DDI with rifampicin)	Buprenorphine	1.39	Hagelberg et al. 2016 (DDI) [53]
iv, 1 mg (30 min)	Buprenorphine	1.43	Mendelson et al. 1997 [62]
iv, 1.2 mg (1 min)	Buprenorphine	1.27	Kuhlman et al. 1996 [63]
iv, 1.2 mg (1 min)	Norbuprenorphine	3.13	Kuhlman et al. 1996 [63]
iv, 2 mg (1 min)	Buprenorphine	1.42	Huestis et al. 2013 (1) [64]
iv, 2 mg (1 min)	Norbuprenorphine	1.54	Huestis et al. 2013 (1) [64]
iv, 4 mg (10 min)	Buprenorphine	1.58	Harris et al. 2000 [64]
iv, 4 mg (1 min)	Buprenorphine	1.40	Huestis et al. 2013 (2) [64]
iv, 4 mg (1 min)	Norbuprenorphine	1.98	Huestis et al. 2013 (2) [64]
iv, 8 mg (1 min)	Buprenorphine	1.44	Huestis et al. 2013 (3) [64]
iv, 8 mg (1 min)	Norbuprenorphine	1.91	Huestis et al. 2013 (3) [64]
iv, 12 mg (1 min)	Buprenorphine	1.46	Huestis et al. 2013 (4) [64]
iv, 12 mg (1 min)	Norbuprenorphine	2.18	Huestis et al. 2013 (4) [64]
iv, 16 mg (1 min)	Buprenorphine	1.40	Huestis et al. 2013 (5) [64]
**Overall MRD**		**1.74 (1.21–4.58)**	
		**34/45 with MRD ≤ 2**	

DDI: drug-drug-interaction, **iv**: intravenous, **m.d.**: multiple dose, **MRD**: mean relative deviation

Table S5: Mean relative deviation (MRD) values of buprenorphine and norbuprenorphine plasma concentration predictions. *(continued)*

Route & Dose	Compound	MRD	Reference
iv, 16 mg (1 min)	Norbuprenorphine	1.99	Huestis et al. 2013 (5) [64]
**MRD**		**1.70 (1.21–3.13)**	
		**20/23 with MRD ≤ 2**	
**Buprenorphine iv children**			
iv, 3 µg/kg (2 min)	Buprenorphine	1.44	Olkkola et al. 1989 (1) [65]
iv, 3 µg/kg (2 min)	Buprenorphine	1.27	Olkkola et al. 1989 (2) [65]
iv, 3 µg/kg (2 min)	Buprenorphine	1.86	Olkkola et al. 1989 (3) [65]
iv, 3 µg/kg (2 min)	Buprenorphine	1.39	Olkkola et al. 1989 (4) [65]
iv, 3 µg/kg (2 min)	Buprenorphine	1.46	Olkkola et al. 1989 (5) [65]
iv, 3 µg/kg (2 min)	Buprenorphine	1.55	Olkkola et al. 1989 (6) [65]
iv, 3 µg/kg (2 min)	Buprenorphine	2.00	Olkkola et al. 1989 (7) [65]
iv, 3 µg/kg (2 min)	Buprenorphine	1.75	Olkkola et al. 1989 (8) [65]
iv, 3 µg/kg (2 min)	Buprenorphine	2.00	Olkkola et al. 1989 (9) [65]
iv, 3 µg/kg (2 min)	Buprenorphine	2.62	Olkkola et al. 1989 (10) [65]
**MRD**		**1.72 (1.27–2.62)**	
		**8/10 with MRD ≤ 2**	
**Buprenorphine iv preterms**			
iv, 3 µg/kg (30 min) 0.72 µg/kg/h (48 h)	Buprenorphine	1.66	Barrett et al. 1993 (1) [30]
iv, 3 µg/kg (30 min) 0.72 µg/kg/h (24 h)	Buprenorphine	1.66	Barrett et al. 1993 (2) [30]
iv, 3 µg/kg (30 min) 0.72 µg/kg/h (11 h)	Buprenorphine	4.58	Barrett et al. 1993 (3) [30]
iv, 3 µg/kg (30 min) 0.72 µg/kg/h (42 h)	Buprenorphine	1.34	Barrett et al. 1993 (4) [30]
iv, 3 µg/kg (30 min) 0.72 µg/kg/h (42 h)	Buprenorphine	1.85	Barrett et al. 1993 (5) [30]
iv, 3 µg/kg (30 min) 1.44 µg/kg/h (23 h)	Buprenorphine	2.65	Barrett et al. 1993 (6) [30]
iv, 3 µg/kg (30 min) 1.44 µg/kg/h (77 h)	Buprenorphine	2.22	Barrett et al. 1993 (7) [30]
iv, 3 µg/kg (30 min) 0.72 µg/kg/h (42 h)	Buprenorphine	2.98	Barrett et al. 1993 (8) [30]
iv, 3 µg/kg (30 min) 2.16 µg/kg/h (81 h)	Buprenorphine	1.46	Barrett et al. 1993 (9) [30]
iv, 3 µg/kg (30 min) 0.72 µg/kg/h (43 h)	Buprenorphine	1.51	Barrett et al. 1993 (10) [30]
iv, 3 µg/kg (30 min) 0.72 µg/kg/h (76 h)	Buprenorphine	1.33	Barrett et al. 1993 (11) [30]
iv, 3 µg/kg (30 min) 0.72 µg/kg/h (118 h)	Buprenorphine	1.42	Barrett et al. 1993 (12) [30]
**MRD**		**1.86 (1.33–4.58)**	
		**8/12 with MRD ≤ 2**	
**Overall MRD**		**1.74 (1.21–4.58)**	
		**34/45 with MRD ≤ 2**	

DDI: drug-drug-interaction, **iv**: intravenous, **m.d.**: multiple dose, **MRD**: mean relative deviation

4.5 Geometric Mean Fold Error (GMFE) of AUC_{last} and C_{max} Predictions

Table S6: Predicted and observed AUC_{last} and C_{max} values of buprenorphine and norbuprenorphine plasma concentrations

		AUC_{last}			C_{max}			
Route	Compound	Pred [ng·h/ml]	Obs [ng·h/ml]	Pred/Obs	Pred [ng/ml]	Obs [ng/ml]	Pred/Obs	Reference
**Buprenorphine iv adults**								
iv, 0.3 mg (2 min)	Buprenorphine	3.34	4.56	0.73	-	-	-	Bai et al. 2016 [58]
iv, 0.3 mg (1 min)	Buprenorphine	1.65	2.59	0.64	-	-	-	Bartlett et al. 1980 [59]
iv, 0.3 mg (1 min)	Buprenorphine	2.75	3.69	0.75	-	-	-	Bullingham et al. 1980 (1) [60]
iv, 0.3 mg (1 min), m.d.	Buprenorphine	3.49	2.99	1.17	-	-	-	Bullingham et al. 1980 (2) [60]
iv, 0.3 mg (1 min)	Buprenorphine	2.14	2.80	0.76	-	-	-	Bullingham et al. 1982 (1) [61]
iv, 0.3 mg (1 min)	Buprenorphine	1.05	1.20	0.87	-	-	-	Bullingham et al. 1982 (2) [61]
iv, 0.3 mg (1 min)	Buprenorphine	1.09	1.56	0.70	-	-	-	Bullingham et al. 1982 (3) [61]
iv, 1 mg (bolus)	Buprenorphine	8.42	11.09	0.76	-	-	-	Hagelberg et al. 2016 [53]
iv, 1 mg (bolus, DDI with rifampicin)	Buprenorphine	7.46	9.41	0.79	-	-	-	Hagelberg et al. 2016 (DDI) [53]
iv, 1 mg (30 min)	Buprenorphine	9.31	10.04	0.93	-	-	-	Mendelson et al. 1997 [62]
iv, 1.2 mg (1 min)	Buprenorphine	18.04	17.20	1.05	-	-	-	Kuhlman et al. 1996 [63]
iv, 1.2 mg (1 min)	Norbuprenorphine	5.98	8.07	0.74	0.49	0.53	0.92	Kuhlman et al. 1996 [63]
iv, 2 mg (1 min)	Buprenorphine	22.97	29.19	0.79	-	-	-	Huestis et al. 2013 (1) [64]
iv, 2 mg (1 min)	Norbuprenorphine	7.67	8.99	0.85	0.73	0.53	1.37	Huestis et al. 2013 (1) [64]
iv, 4 mg (10 min)	Buprenorphine	51.44	51.64	1.00	-	-	-	Harris et al. 2000 [64]
iv, 4 mg (1 min)	Buprenorphine	47.68	60.38	0.79	-	-	-	Huestis et al. 2013 (2) [64]
iv, 4 mg (1 min)	Norbuprenorphine	15.34	13.12	1.17	1.46	0.94	1.56	Huestis et al. 2013 (2) [64]
iv, 8 mg (1 min)	Buprenorphine	95.68	115.63	0.83	-	-	-	Huestis et al. 2013 (3) [64]
iv, 8 mg (1 min)	Norbuprenorphine	30.68	26.68	1.15	2.91	1.82	1.61	Huestis et al. 2013 (3) [64]
iv, 12 mg (1 min)	Buprenorphine	143.30	179.05	0.80	-	-	-	Huestis et al. 2013 (4) [64]
iv, 12 mg (1 min)	Norbuprenorphine	46.05	38.98	1.18	4.37	2.91	1.50	Huestis et al. 2013 (4) [64]
iv, 16 mg (1 min)	Buprenorphine	191.08	201.30	0.95	-	-	-	Huestis et al. 2013 (5) [64]
iv, 16 mg (1 min)	Norbuprenorphine	61.44	57.87	1.06	5.84	3.53	1.65	Huestis et al. 2013 (5) [64]
**GMFE**				**1.22 (1.00–1.57)**			**1.45 (1.09–1.65)**	
				**23/23 with GMFE ≤ 2**			**6/6 with GMFE ≤ 2**	
**Buprenorphine iv children**								
iv, 3 µg/kg (2 min)	Buprenorphine	0.92	1.26	0.73	-	-	-	Olkkola et al. 1989 (1) [65]
iv, 3 µg/kg (2 min)	Buprenorphine	0.79	0.66	1.21	-	-	-	Olkkola et al. 1989 (2) [65]
iv, 3 µg/kg (2 min)	Buprenorphine	0.79	0.43	1.83	-	-	-	Olkkola et al. 1989 (3) [65]
iv, 3 µg/kg (2 min)	Buprenorphine	0.55	0.43	1.28	-	-	-	Olkkola et al. 1989 (4) [65]
iv, 3 µg/kg (2 min)	Buprenorphine	0.55	0.41	1.36	-	-	-	Olkkola et al. 1989 (5) [65]
iv, 3 µg/kg (2 min)	Buprenorphine	0.56	0.39	1.44	-	-	-	Olkkola et al. 1989 (6) [65]
iv, 3 µg/kg (2 min)	Buprenorphine	0.55	0.28	1.97	-	-	-	Olkkola et al. 1989 (7) [65]
iv, 3 µg/kg (2 min)	Buprenorphine	0.55	0.37	1.49	-	-	-	Olkkola et al. 1989 (8) [65]
iv, 3 µg/kg (2 min)	Buprenorphine	0.34	0.23	1.45	-	-	-	Olkkola et al. 1989 (9) [65]
iv, 3 µg/kg (2 min)	Buprenorphine	0.33	0.14	2.31	-	-	-	Olkkola et al. 1989 (10) [65]
**GMFE**				**1.54 (1.21–2.31)**				
				**9/10 with GMFE ≤ 2**				
**Buprenorphine iv preterms**								
iv, 3 µg/kg (30 min) 0.72 µg/kg/h (48 h)	Buprenorphine	93.54	67.34	1.39	2.74	3.02	0.91	Barrett et al. 1993 (1) [30]
iv, 3 µg/kg (30 min) 0.72 µg/kg/h (24 h)	Buprenorphine	26.99	31.77	0.85	2.15	2.80	0.77	Barrett et al. 1993 (2) [30]
iv, 3 µg/kg (30 min) 0.72 µg/kg/h (11 h)	Buprenorphine	18.81	4.71	3.99	2.99	0.73	4.10	Barrett et al. 1993 (3) [30]
**Overall GMFE**				**1.37 (1.00–3.99)**			**1.45 (1.02–4.10)**	
				**41/45 with GMFE ≤ 2**			**16/18 with GMFE ≤ 2**	

-: not calculated, **DDI**: drug-drug-interaction, **GMFE**: geometric mean fold error, **iv**: intravenous, **m.d.**: multiple dose, **obs**: observed, **pred**: predicted

Table S6: Predicted and observed AUC_{last} and C_{max} values of buprenorphine and norbuprenorphine plasma concentrations *(continued)*

Route	Compound	Pred [ng·h/ml]	Obs [ng·h/ml]	Pred/Obs	Pred [ng/ml]	Obs [ng/ml]	Pred/Obs	Reference
iv, 3 µg/kg (30 min) 0.72 µg/kg/h (42 h)	Buprenorphine	120.02	95.53	1.26	2.76	2.29	1.21	Barrett et al. 1993 (4) [30]
iv, 3 µg/kg (30 min) 0.72 µg/kg/h (42 h)	Buprenorphine	85.51	134.06	0.64	2.88	4.55	0.63	Barrett et al. 1993 (5) [30]
iv, 3 µg/kg (30 min) 1.44 µg/kg/h (23 h)	Buprenorphine	66.15	28.34	2.33	4.64	3.20	1.45	Barrett et al. 1993 (6) [30]
iv, 3 µg/kg (30 min) 1.44 µg/kg/h (77 h)	Buprenorphine	322.93	220.52	1.46	5.34	4.46	1.20	Barrett et al. 1993 (7) [30]
iv, 3 µg/kg (30 min) 0.72 µg/kg/h (42 h)	Buprenorphine	99.31	230.04	0.43	2.05	4.17	0.49	Barrett et al. 1993 (8) [30]
iv, 3 µg/kg (30 min) 2.16 µg/kg/h (81 h)	Buprenorphine	797.92	583.00	1.37	10.17	10.42	0.98	Barrett et al. 1993 (9) [30]
iv, 3 µg/kg (30 min) 0.72 µg/kg/h (43 h)	Buprenorphine	132.68	99.25	1.34	2.74	2.50	1.10	Barrett et al. 1993 (10) [30]
iv, 3 µg/kg (30 min) 0.72 µg/kg/h (76 h)	Buprenorphine	238.11	209.79	1.14	3.05	3.33	0.92	Barrett et al. 1993 (11) [30]
iv, 3 µg/kg (30 min) 0.72 µg/kg/h (118 h)	Buprenorphine	485.01	528.79	0.92	4.47	7.59	0.59	Barrett et al. 1993 (12) [30]
**GMFE**				**1.57 (1.09–3.99)**			**1.44 (1.02–4.10)**	
				**9/12 with GMFE ≤ 2**			**10/12 with GMFE ≤ 2**	
**Overall GMFE**				**1.37 (1.00–3.99)**			**1.45 (1.02–4.10)**	
				**41/45 with GMFE ≤ 2**			**16/18 with GMFE ≤ 2**	

-: not calculated, **DDI**: drug-drug-interaction, **GMFE**: geometric mean fold error, **iv**: intravenous, **m.d.**: multiple dose, **obs**: observed, **pred**: predicted

4.6 Buprenorphine and Norbuprenorphine PBPK Model Sensitivity Analysis

A sensitivity analysis of the buprenorphine and norbuprenorphine PBPK models (adults and pediatrics) to single parameter changes (local sensitivity analysis) was performed. Sensitivities of the PBPK models were calculated as the relative changes of the predicted AUCs extrapolated to infinity (AUC_{inf}) of buprenorphine and norbuprenorphine, respectively, to the relative variation of model input parameters in a steady-state scenario (1.4 mg (adults), 0.7 mg (children), 0.009 mg (preterm neonates), 168 hours long-term infusion, mimicking steady-state plasma concentrations of about 0.13 ng/ml, which were achieved with an administration of marketed transdermal buprenorphine patches [56]). Parameters, optimized as well as parameters fixed to literature values, were included into the analysis if they had significant impact in former models (e.g. glomerular filtration rate fraction, maximum reaction velocity, inhibition constants), if they might have a strong influence due to calculation methods used in the model (e.g. lipophilicity) and/or if they have been optimized. The analyses were performed using a relative perturbation of parameters of 10%. Model sensitivity to a model parameter was calculated as follows:

$$S = \frac{\Delta AUC_{inf}}{\Delta p} \cdot \frac{p}{AUC_{inf}} \qquad \text{(S18)}$$

where S is the sensitivity of the AUC_{inf} to the examined model parameter, ΔAUC_{inf} is the change of the AUC_{inf}, AUC_{inf} is the simulated AUC_{inf} with the original parameter value, p is the original model parameter value and Δp is the variation of the model parameter value. A sensitivity value of +1.0 signifies that a 10% increase of the examined parameter causes a 10 % increase of the simulated AUC_{inf}.

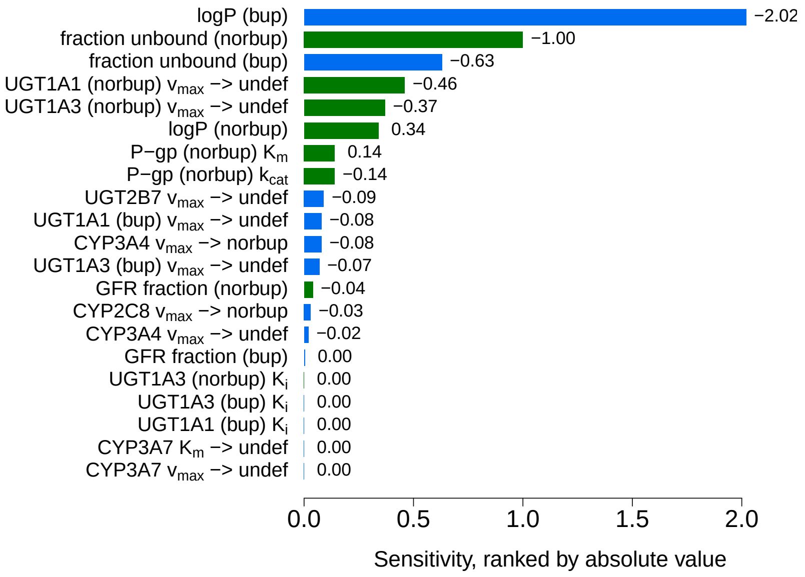

Figure S9: Sensitivity analysis of the adult PBPK model for buprenorphine and norbuprenorphine. Sensitivity of the model to single parameters, calculated as change of the simulated buprenorphine (blue) and norbuprenorphine (green) AUC_{inf} following a 168 hours long-term infusion, mimicking steady-state plasma concentrations of about 0.13 ng/ml, which were achieved with an administration of marketed transdermal buprenorphine patches in adults [56]. **bup**: buprenorphine, **GFR**: glomerular filtration rate, $\mathbf{k_{cat}}$: transport rate constant (turnover number), $\mathbf{K_i}$: concentration for half-maximal inhibition, $\mathbf{K_m}$: Michaelis-Menten constant, **norbup**: norbuprenorphine, **P-gp**: P-glycoprotein, **undef**: undefined metabolite, $\mathbf{v_{max}}$: maximum reaction velocity

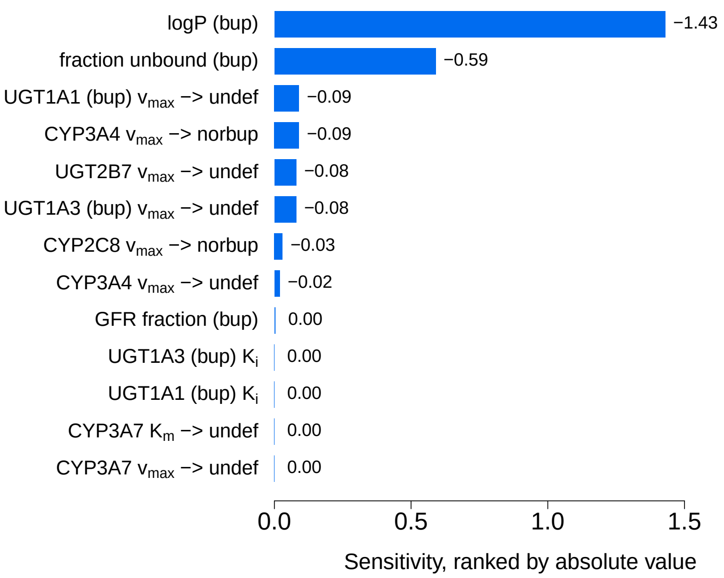

Figure S10: Sensitivity analysis of the PBPK model in children for buprenorphine. Sensitivity of the model to single parameters, calculated as change of the simulated buprenorphine (blue) AUC_{inf} following a 168 hours long-term infusion, mimicking steady-state plasma concentrations of about 0.13 ng/ml, which were achieved with an administration of marketed transdermal buprenorphine patches in adults [56]. **bup**: buprenorphine, **GFR**: glomerular filtration rate, $\mathbf{K_i}$: concentration for half-maximal inhibition, $\mathbf{K_m}$: Michaelis-Menten constant, **norbup**: norbuprenorphine, **undef**: undefined metabolite, $\mathbf{v_{max}}$: maximum reaction velocity

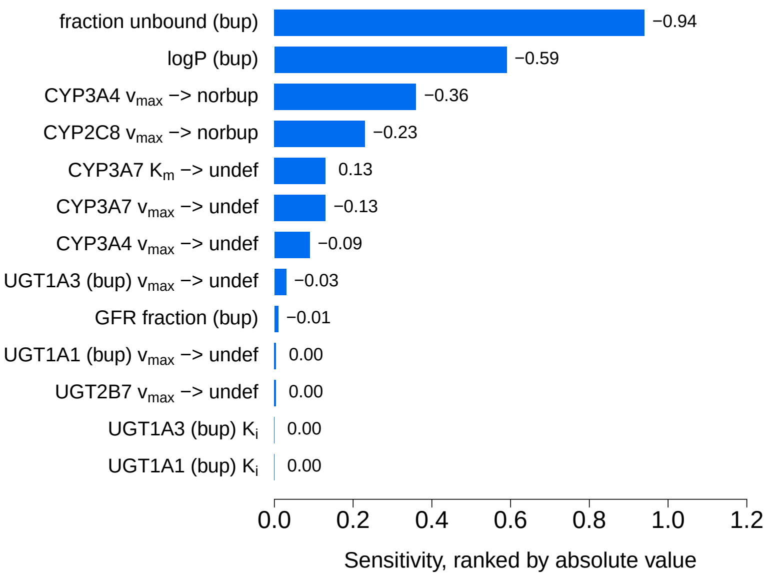

Figure S11: Sensitivity analysis of the PBPK model in pediatrics for buprenorphine. Sensitivity of the model to single parameters, calculated as change of the simulated buprenorphine AUC_{inf} following a 168 hours long-term infusion, mimicking steady-state plasma concentrations of about 0.13 ng/ml, which were achieved with an administration of marketed transdermal buprenorphine patches in adults [56]. **bup**: buprenorphine, **GFR**: glomerular filtration rate, $\mathbf{K_i}$: concentration for half-maximal inhibition, $\mathbf{K_m}$: Michaelis-Menten constant, **norbup**: norbuprenorphine, **undef**: undefined metabolite, $\mathbf{v_{max}}$: maximum reaction velocity

References

[1] Maharaj AR, Barrett JS, Edginton AN (2013) A workflow example of PBPK modeling to support pediatric research and development: case study with lorazepam. The AAPS journal 15(2):455–64

[2] Maharaj AR, Edginton AN (2014) Physiologically Based Pharmacokinetic Modeling and Simulation in Pediatric Drug Development. CPT: Pharmacometrics & Systems Pharmacology 3(11):1–13

[3] Leong R, Vieira MLT, Zhao P, Mulugeta Y, Lee CS, Huang SM, Burckart GJ (2012) Regulatory experience with physiologically based pharmacokinetic modeling for pediatric drug trials. Clinical pharmacology and therapeutics 91(5):926–31

[4] Ince I, Solodenko J, Frechen S, Dallmann A, Niederalt C, Schlender J, Burghaus R, Lippert J, Willmann S (2019) Predictive Pediatric Modeling and Simulation Using Ontogeny Information. The Journal of Clinical Pharmacology 59(S1):S95–S103

[5] Picard N, Cresteil T, Djebli N, Marquet P (2005) In vitro metabolism study of buprenorphine: evidence for new metabolic pathways. Drug metabolism and disposition: the biological fate of chemicals 33(5):689–95

[6] Chang Y, Moody DE (2009) Glucuronidation of buprenorphine and norbuprenorphine by human liver microsomes and UDP-glucuronosyltransferases. Drug metabolism letters 3(2):101–7

[7] Brown SM, Campbell SD, Crafford A, Regina KJ, Holtzman MJ, Kharasch ED (2012) P-glycoprotein is a major determinant of norbuprenorphine brain exposure and antinociception. The Journal of pharmacology and experimental therapeutics 343(1):53–61

[8] Oechsler S, Skopp G (2010) An in vitro approach to estimate putative inhibition of buprenorphine and norbuprenorphine glucuronidation. International Journal of Legal Medicine 124(3):187–194

[9] Obach RS (1997) Nonspecific binding to microsomes: impact on scale-up of in vitro intrinsic clearance to hepatic clearance as assessed through examination of warfarin, imipramine, and propranolol. Drug metabolism and disposition: the biological fate of chemicals 25(12):1359–69

[10] Moody DE, Slawson MH, Strain EC, Laycock JD, Spanbauer AC, Foltz RL (2002) A Liquid Chromatographic-Electrospray Ionization-Tandem Mass Spectrometric Method for Determination of Buprenorphine, Its Metabolite, norBuprenorphine, and a Coformulant, Naloxone, That Is Suitable for in Vivo and in Vitro Metabolism Studies. Analytical Biochemistry 306(1):31–39

[11] Williams JA, Ring BJ, Cantrell VE, Jones DR, Eckstein J, Ruterbories K, Hamman MA, Hall SD, Wrighton SA (2002) Comparative Metabolic Capabilities of CYP3A4, CYP3A5, and CYP3A7. Drug Metabolism and Disposition 30(8):883–891

[12] Niwa T, Okamoto A, Narita K, Toyota M, Kato K, Kobayashi K, Sasaki S (2020) Comparison of steroid hormone hydroxylation mediated by cytochrome P450 3A subfamilies. Archives of Biochemistry and Biophysics 682:108283

[13] Hindmarsh A, Reynolds D, Serban R, Woodward C, Gardner DJ, Cohen S, Taylor A, Peles S, Banks L, Shumaker D (2018) Open Systems Pharmacology Suite Manual, Version 7.4

[14] Rodrigues AD (1999) Integrated cytochrome P450 reaction phenotyping: attempting to bridge the gap between cDNA-expressed cytochromes P450 and native human liver microsomes. Biochemical pharmacology 57(5):465–80

[15] Stevens JC, Hines RN, Gu C, Koukouritaki SB, Manro JR, Tandler PJ, Zaya MJ (2003) Developmental Expression of the Major Human Hepatic CYP3A Enzymes. Journal of Pharmacology and Experimental Therapeutics 307(2):573–582

[16] Everhart E, Cheung P, Mendelson J, Upton R, Jones R (1999) The mass balance of buprenorphine in humans. Clinical Pharmacology & Therapeutics 65(2):152–152

[17] Chiang CN, Hawks RL (2003) Pharmacokinetics of the combination tablet of buprenorphine and naloxone. Drug and alcohol dependence 70(2 Suppl):S39–47

[18] Willmann S, Höhn K, Edginton A, Sevestre M, Solodenko J, Weiss W, Lippert J, Schmitt W (2007) Development of a physiology-based whole-body population model for assessing the influence of individual variability on the pharmacokinetics of drugs. Journal of Pharmacokinetics and Pharmacodynamics 34(3):401–431

[19] Valentin J (2002) Basic anatomical and physiological data for use in radiological protection: reference values. Annals of the ICRP 32(3-4):1–277

[20] National Center for Health Statistics (1997) Third National Health and Nutrition Examination Survey (NHANES III). Tech. rep., Hyattsville, MD 20782

[21] Nishimura M, Yaguti H, Yoshitsugu H, Naito S, Satoh T (2003) Tissue distribution of mRNA expression of human cytochrome P450 isoforms assessed by high-sensitivity real-time reverse transcription PCR. Journal of the Pharmaceutical Society of Japan 123(5):369–75

[22] Nishimura M, Naito S (2005) Tissue-specific mRNA Expression Profiles of Human ATP-binding Cassette and Solute Carrier Transporter Superfamilies. Drug Metabolism and Pharmacokinetics 20(6):452–477

[23] Nishimura M, Naito S (2006) Tissue-Specific mRNA Expression Profiles of Human Phase I Metabolizing Enzymes Except for Cytochrome P450 and Phase II Metabolizing Enzymes. Drug Metabolism and Pharmacokinetics 21(5):357–374

[24] Open Systems Pharmacology Suite Community (2018) PK-Sim® Ontogeny Database Documentation, Version 7.3. https://github.com/Open-Systems-Pharmacology/OSPSuite.Documentation/blob/master/PK-SimOntogenyDatabaseVersion7.3.pdf, accessed: 2020-03-25

[25] Margaillan G, Rouleau M, Klein K, Fallon JK, Caron P, Villeneuve L, Smith PC, Zanger UM, Guillemette C (2015) Multiplexed Targeted Quantitative Proteomics Predicts Hepatic Glucuronidation Potential. Drug Metabolism and Disposition 43(9):1331–1335

[26] National Center for Biotechnology Information (NCBI) (2010) Expressed Sequence Tags (EST) from UniGene.

[27] Hanke N, Frechen S, Moj D, Britz H, Eissing T, Wendl T, Lehr T (2018) PBPK Models for CYP3A4 and P-gp DDI Prediction: A Modeling Network of Rifampicin, Itraconazole, Clarithromycin, Midazolam, Alfentanil, and Digoxin. CPT: Pharmacometrics and Systems Pharmacology 7(10):647–659

[28] Prasad B, Evers R, Gupta A, Hop CECA, Salphati L, Shukla S, Ambudkar SV, Unadkat JD (2014) Interindividual Variability in Hepatic Organic Anion-Transporting Polypeptides and P-Glycoprotein (ABCB1) Protein Expression: Quantification by Liquid Chromatography Tandem Mass Spectroscopy and Influence of Genotype, Age, and Sex. Drug Metabolism and Disposition 42(1):78–88

[29] McCarver DG, Hines RN (2002) The ontogeny of human drug-metabolizing enzymes: phase II conjugation enzymes and regulatory mechanisms. The Journal of pharmacology and experimental therapeutics 300(2):361–6

[30] Barrett D, Simpson J, Rutter N, Kurihara-Bergstrom T, Shaw P, Davis S (1993) The pharmacokinetics and physiological effects of buprenorphine infusion in premature neonates. British Journal of Clinical Pharmacology 36(3):215–219

[31] Kajosaari LI, Laitila J, Neuvonen PJ, Backman JT (2005) Metabolism of repaglinide by CYP2C8 and CYP3A4 in vitro: effect of fibrates and rifampicin. Basic & clinical pharmacology & toxicology 97(4):249–56

[32] Rajaonarison J, Lacarelle B, Catalin J, Placidi M, Rahmani R (1992) 3'-azido-3'-deoxythymidine drug interactions. Screening for inhibitors in human liver microsomes. Drug Metab Dispos 20(4):578–584

[33] Chiou WJ, de Morais SM, Kikuchi R, Voorman RL, Li X, Bow DAJ (2014) In vitro OATP1B1 and OATP1B3 inhibition is associated with observations of benign clinical unconjugated hyperbilirubinemia. Xenobiotica 44(3):276–282

[34] Soars MG, Petullo DM, Eckstein JA, Kasper SC, Wrighton SA (2004) An assessment of udp-glucuronosyltransferase induction using primary human hepatocytes. Drug metabolism and disposition: the biological fate of chemicals 32(1):140–8

[35] Trottier J, El Husseini D, Perreault M, Pâquet S, Caron P, Bourassa S, Verreault M, Inaba TT, Poirier GG, Bélanger A, Guillemette C, Trauner M, Barbier O (2010) The Human UGT1A3 Enzyme Conjugates Norursodeoxycholic Acid into a C 23 -ester Glucuronide in the Liver. Journal of Biological Chemistry 285(2):1113–1121

[36] Templeton IE, Houston JB, Galetin A (2011) Predictive Utility of In Vitro Rifampin Induction Data Generated in Fresh and Cryopreserved Human Hepatocytes, Fa2N-4, and HepaRG Cells. Drug Metabolism and Disposition 39(10):1921–1929

[37] Reitman ML, Chu X, Cai X, Yabut J, Venkatasubramanian R, Zajic S, Stone JA, Ding Y, Witter R, Gibson C, Roupe K, Evers R, Wagner JA, Stoch A (2011) Rifampin's Acute Inhibitory and Chronic Inductive Drug Interactions: Experimental and Model-Based Approaches to Drug–Drug Interaction Trial Design. Clinical Pharmacology & Therapeutics 89(2):234–242

[38] Greiner B, Eichelbaum M, Fritz P, Kreichgauer HP, von Richter O, Zundler J, Kroemer HK (1999) The role of intestinal P-glycoprotein in the interaction of digoxin and rifampin. Journal of Clinical Investigation 104(2):147–153

[39] Buckley DB, Wiegand CM, Prentiss PL, Fahmi OA (2013) Time-course of cytochrome P450 (CYP450) induction in cultured human hepatocytes: Evaluation of activity and mRNA expression profiles for six inducible CYP450 enzymes. ISSX.

[40] Wishart DS, Knox C, Guo AC, Shrivastava S, Hassanali M, Stothard P, Chang Z, Woolsey J (2006) DrugBank: a comprehensive resource for in silico drug discovery and exploration. Nucleic acids research 34(Database issue):D668–72

[41] Merck Research Laboratories (2006) The Merck Index 14th edition: Rifampin. Merck & Co., Inc., Whitehouse Station, NJ, USA

[42] Boman G, Ringberger VA (1974) Binding of rifampicin by human plasma proteins. European journal of clinical pharmacology 7(5):369–73

[43] Baneyx G, Parrott N, Meille C, Iliadis A, Lavé T (2014) Physiologically based pharmacokinetic modeling of CYP3A4 induction by rifampicin in human: influence of time between substrate and inducer administration. European journal of pharmaceutical sciences : official journal of the European Federation for Pharmaceutical Sciences 56:1–15

[44] Loos U, Musch E, Jensen JC, Mikus G, Schwabe HK, Eichelbaum M (1985) Pharmacokinetics of oral and intravenous rifampicin during chronic administration. Klinische Wochenschrift 63(23):1205–11

[45] Tirona RG, Leake BF, Wolkoff AW, Kim RB (2003) Human organic anion transporting polypeptide-C (SLC21A6) is a major determinant of rifampin-mediated pregnane X receptor activation. The Journal of pharmacology and experimental therapeutics 304(1):223–8

[46] Nakajima A, Fukami T, Kobayashi Y, Watanabe A, Nakajima M, Yokoi T (2011) Human arylacetamide deacetylase is responsible for deacetylation of rifamycins: rifampicin, rifabutin, and rifapentine. Biochemical pharmacology 82(11):1747–56

[47] Collett A, Tanianis-Hughes J, Hallifax D, Warhurst G (2004) Predicting P-glycoprotein effects on oral absorption: correlation of transport in Caco-2 with drug pharmacokinetics in wild-type and mdr1a(-/-) mice in vivo. Pharmaceutical research 21(5):819–26

[48] Shou M, Hayashi M, Pan Y, Xu Y, Morrissey K, Xu L, Skiles GL (2008) Modeling, prediction, and in vitro in vivo correlation of CYP3A4 induction. Drug metabolism and disposition: the biological fate of chemicals 36(11):2355–70

[49] Hirano M, Maeda K, Shitara Y, Sugiyama Y (2006) Drug-drug interaction between pitavastatin and various drugs via OATP1B1. Drug metabolism and disposition: the biological fate of chemicals 34(7):1229–36

[50] Annaert P, Ye ZW, Stieger B, Augustijns P (2010) Interaction of HIV protease inhibitors with OATP1B1, 1B3, and 2B1. Xenobiotica; the fate of foreign compounds in biological systems 40(3):163–76

[51] Rodgers T, Leahy D, Rowland M (2005) Physiologically based pharmacokinetic modeling 1: Predicting the tissue distribution of moderate-to-strong bases. Journal of Pharmaceutical Sciences 94(6):1259–1276

[52] Rodgers T, Rowland M (2006) Physiologically based pharmacokinetic modelling 2: Predicting the tissue distribution of acids, very weak bases, neutrals and zwitterions. Journal of Pharmaceutical Sciences 95(6):1238 – 1257

[53] Hagelberg NM, Fihlman M, Hemmilä T, Backman JT, Laitila J, Neuvonen PJ, Laine K, Olkkola KT, Saari TI (2016) Rifampicin decreases exposure to sublingual buprenorphine in healthy subjects. Pharmacology Research & Perspectives 4(6):e00271

[54] Tod M, Jullien V, Pons G (2008) Facilitation of Drug Evaluation in Children by Population Methods and Modelling†. Clinical Pharmacokinetics 47(4):231–243

[55] Mahmood I, Tegenge MA (2019) A Comparative Study Between Allometric Scaling and Physiologically Based Pharmacokinetic Modeling for the Prediction of Drug Clearance From Neonates to Adolescents. The Journal of Clinical Pharmacology 59(2):189–197

[56] Kapil RP, Cipriano A, Friedman K, Michels G, Shet MS, Colucci SV, Apseloff G, Kitzmiller J, Harris SC (2013) Once-Weekly Transdermal Buprenorphine Application Results in Sustained and Consistent Steady-State Plasma Levels. Journal of Pain and Symptom Management 46(1):65–75

[57] Edginton AN, Schmitt W, Voith B, Willmann S (2006) A Mechanistic Approach for the Scaling of Clearance in Children. Clinical Pharmacokinetics 45(7):683–704

[58] Bai SA, Xiang Q, Finn A (2016) Evaluation of the Pharmacokinetics of Single- and Multiple-dose Buprenorphine Buccal Film in Healthy Volunteers. Clinical Therapeutics 38(2):358–369

[59] Bartlett AJ, Lloyd-Jones JG, Rance MJ, Flockhart IR, Dockray GJ, Bennett MR, Moore RA (1980) The radioimmunoassay of buprenorphine. European Journal of Clinical Pharmacology 18(4):339–345

[60] Bullingham RE, McQuay HJ, Moore A, Bennett MR (1980) Buprenorphine kinetics. Clinical pharmacology and therapeutics 28(5):667–72

[61] Bullingham RE, McQuay HJ, Porter EJ, Allen MC, Moore RA (1982) Sublingual buprenorphine used postoperatively: ten hour plasma drug concentration analysis. British journal of clinical pharmacology 13(5):665–73

[62] Mendelson J, Upton RA, Everhart ET, Jacob III P, Jones RT (1997) Bioavailability of Sublingual Buprenorphine. The Journal of Clinical Pharmacology 37(1):31–37

[63] Kuhlman JJ, Lalani S, Magluilo J, Levine B, Darwin WD, Johnson RE, Cone EJ (1996) Human Pharmacokinetics of Intravenous, Sublingual, and Buccal Buprenorphine. Journal of Analytical Toxicology 20(6):369–378

[64] Huestis M, Cone E, Pirnay S, Umbricht A, Preston K (2013) Intravenous buprenorphine and norbuprenorphine pharmacokinetics in humans. Drug and Alcohol Dependence 131(3):258–262

[65] Olkkola K, Maunuksela E, Korpela R (1989) Pharmacokinetics of intravenous buprenorphine in children. British Journal of Clinical Pharmacology 28(2):202–204
